# SPON2 facilitates osteosarcoma development by inducing M2 macrophage polarization through activation of the NF-κB/VEGF signaling axis

**DOI:** 10.1038/s41420-025-02626-2

**Published:** 2025-07-29

**Authors:** Xinchang Lu, Xueping Zhang, Fengzhen Zhang, Wenhao Wang, Ruijie Liu, Yubao Hou, Weiye Shi, Jiazhen Li, Changliang Peng

**Affiliations:** 1 Department of Orthopedic Surgery, The First Affiliated Hospital of Zhengzhou University, Zhengzhou, China; 2 Department of Magnetic Resonance Imaging, The First Affiliated Hospital of Zhengzhou University, Zhengzhou, China; 3 Department of Spinal Surgery, The Second Hospital of Shandong University, Shandong University, Jinan, China

**Keywords:** Bone cancer, Tumour immunology

## Abstract

Osteosarcoma (OS) is an aggressive bone tumor with poor prognosis, particularly in metastatic cases. Here, we identify spondin 2 (SPON2) as a key driver of OS progression. SPON2 is significantly upregulated in OS tissues and cell lines and correlates with shorter patient survival. Functional assays show that SPON2 promotes OS cell proliferation, migration, invasion, and angiogenesis by enhancing the secretion of IL10, CCL2, and CSF1, which leads to M2 macrophage polarization and an immunosuppressive tumor microenvironment. In vitro, SPON2 knockdown reduces M2 macrophage markers and attenuates EMT phenotypes, as evidenced by decreased mesenchymal markers and preserved epithelial characteristics. Mechanistically, SPON2 activates the NF-κB/VEGF signaling axis to drive both macrophage polarization and EMT, thereby promoting tumor progression. In vivo, SPON2 knockdown in OS xenografts suppresses tumor growth, lung metastasis, and M2 polarization, while increasing M1-associated markers. Lipopolysaccharide (LPS) stimulation restores cytokine secretion and EMT marker expression in SPON2-knockdown models, suggesting that SPON2 acts through inflammation-responsive pathways. Together, these findings establish SPON2 as a key regulator of both immune modulation and metastatic behavior in OS, and highlight its potential as a therapeutic target.

## Introduction

Osteosarcoma (OS) is a highly malignant primary bone tumor that predominantly affects adolescents and young adults. It is characterized by rapid growth, early pulmonary metastasis, and poor clinical outcomes [1]. Despite advances in neoadjuvant chemotherapy and surgical techniques, the overall 5-year survival rate for OS patients remains relatively low, especially for those with metastatic disease [2, 3]. Lung metastasis is the most common and life-threatening complication of OS, and significantly worsens prognosis [4]. These challenges highlight the urgent need for more effective therapeutic strategies to prevent and treat metastasis and improve patient outcomes.

Tumor-associated macrophages (TAMs) are a major component of the tumor microenvironment (TME) and have been shown to play a critical role in cancer progression [5], including in OS [6]. TAMs exhibit phenotypic plasticity and are generally classified into classically activated M1 macrophages and alternatively activated M2 macrophages. M1 macrophages exert anti-tumor effects by producing pro-inflammatory cytokines and promoting immune surveillance, whereas M2 macrophages support tumor growth by enhancing angiogenesis, invasion, metastasis, and immunosuppression [7, 8]. In OS, the accumulation of M2-polarized TAMs has been linked to tumor aggressiveness and resistance to therapy [9–11]. As a result, modulating macrophage polarization has emerged as a promising approach for therapeutic intervention.

Spondin2 (SPON2), also known as M-spondin or DIL1, is a secreted extracellular matrix protein belonging to the Mindin-F-spondin (FS) family [12]. It contains an N-terminal FS domain responsible for integrin binding and a C-terminal thrombospondin type 1 repeat (TSR) domain, that interacts with lipopolysaccharide (LPS), with mannose modification enhancing this interaction [12]. These domains allow SPON2 to engage both innate and adaptive immune pathways [12–15]. As a ligand for macrophage antigen 1 (Mac-1), SPON2 activates spleen tyrosine kinase (SYK) and induces nuclear factor-κB (NF-κB) p65 nuclear translocation, promoting phagocytosis and inflammatory signaling [16, 17]. SPON2 also facilitates dendritic cell maturation, T-cell activation, and responses to bacterial and viral infections [13, 18]. Notably, SPON2 is highly expressed in multiple solid tumors, including breast [19], gastric [20], lung [21, 22], prostate [23], colorectal [22], and liver cancers [24], where it contributes to tumorigenesis by enhancing epithelial-mesenchymal transition (EMT), angiogenesis, immune evasion, and macrophage polarization [25, 26]. However, its role and underlying mechanism in OS remain poorly understood.

In this study, we demonstrate that SPON2 is significantly upregulated in OS tissues and cell lines and is associated with poor prognosis. Functional experiments reveal that SPON2 promotes OS cell proliferation, migration, and metastasis. Mechanistically, SPON2 enhances interleukin 10 (IL10), C-C motif chemokine ligand 2 (CCL2), and colony stimulating factor 1 (CSF1) secretion, drives M2 macrophage polarization, and activates the NF-κB/VEGF signaling axis to facilitate EMT and tumor progression. These findings suggest that SPON2 plays an important role in regulating macrophage activity within the immunosuppressive tumor microenvironment of OS and may serve as a promising therapeutic target.

## Results

### SPON2 is upregulated in OS and correlates with poor prognosis

**Fig. 1:**
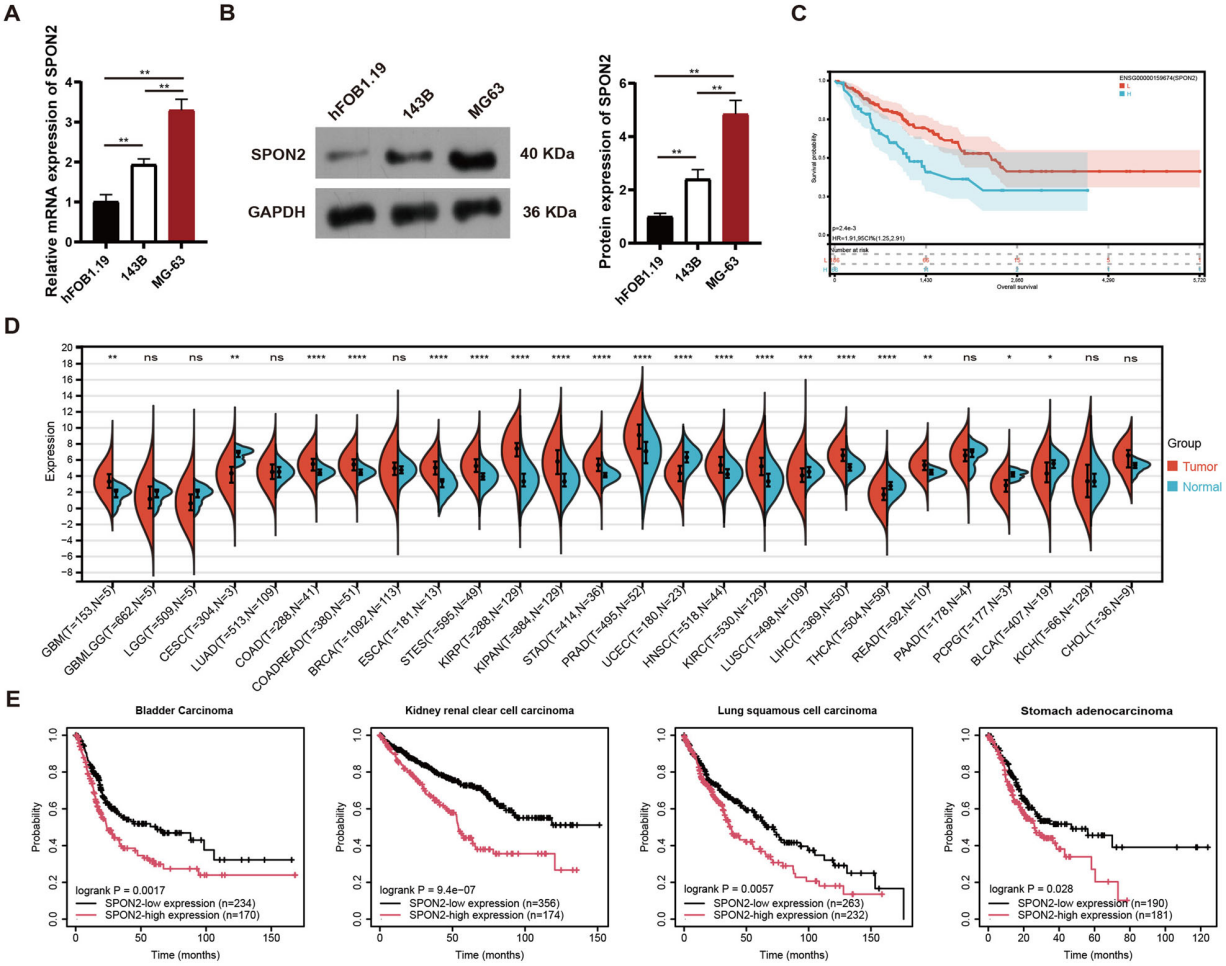
SPON2 expression is upregulated in OS and correlates with poor prognosis. **A, B** mRNA and protein expression of SPON2 in OS cell lines (MG63 and 143B) compared to the normal osteoblast cell line hFOB1.19. Data show a significant increase in SPON2 expression in OS cells. Data are shown as mean ± SD (*n* = 3 biological replicates). Statistical analysis was performed using one-way ANOVA followed by Tukey’s post-hoc test. **p < 0.01. **C** Survival analysis indicates that higher SPON2 expression correlates with shorter overall survival in OS patients. **D** The mRNA expression of SPON2 in tumor and normal tissue samples was examined by analyzing TCGA database. The Unpaired Wilcoxon Rank Sum and Signed Rank Tests were employed. *p < 0.05, **p < 0.01, ***p < 0.001, ****p < 0.0001. ns: not significant. **E** Survival analysis of SPON2 in TCGA dataset.

To investigate the role of SPON2 in the progression of OS, we analyzed its mRNA and protein expression levels in two OS cell lines and one normal osteoblast cell line (hFOB1.19). We found that both mRNA and protein levels of SPON2 were significantly elevated in OS cells compared to hFOB1.19 cells (Fig. 1A, B), indicating a potential involvement of SPON2 in OS progression. To further examine the relevance of SPON2 in OS development, we utilized data from The Cancer Genome Atlas (TCGA). Survival analysis demonstrated that higher SPON2 expression was associated with shorter overall survival in OS patients (Fig. 1C). Moreover, SPON2 expression was consistently elevated across various solid tumors and was correlated with poorer prognosis (Fig. 1D, E). These findings collectively suggest that SPON2 may play a pivotal role in OS progression and could be involved in the development of other solid tumors.

### SPON2 drives OS development and metastasis through regulation of key signaling pathways

**Fig. 2:**
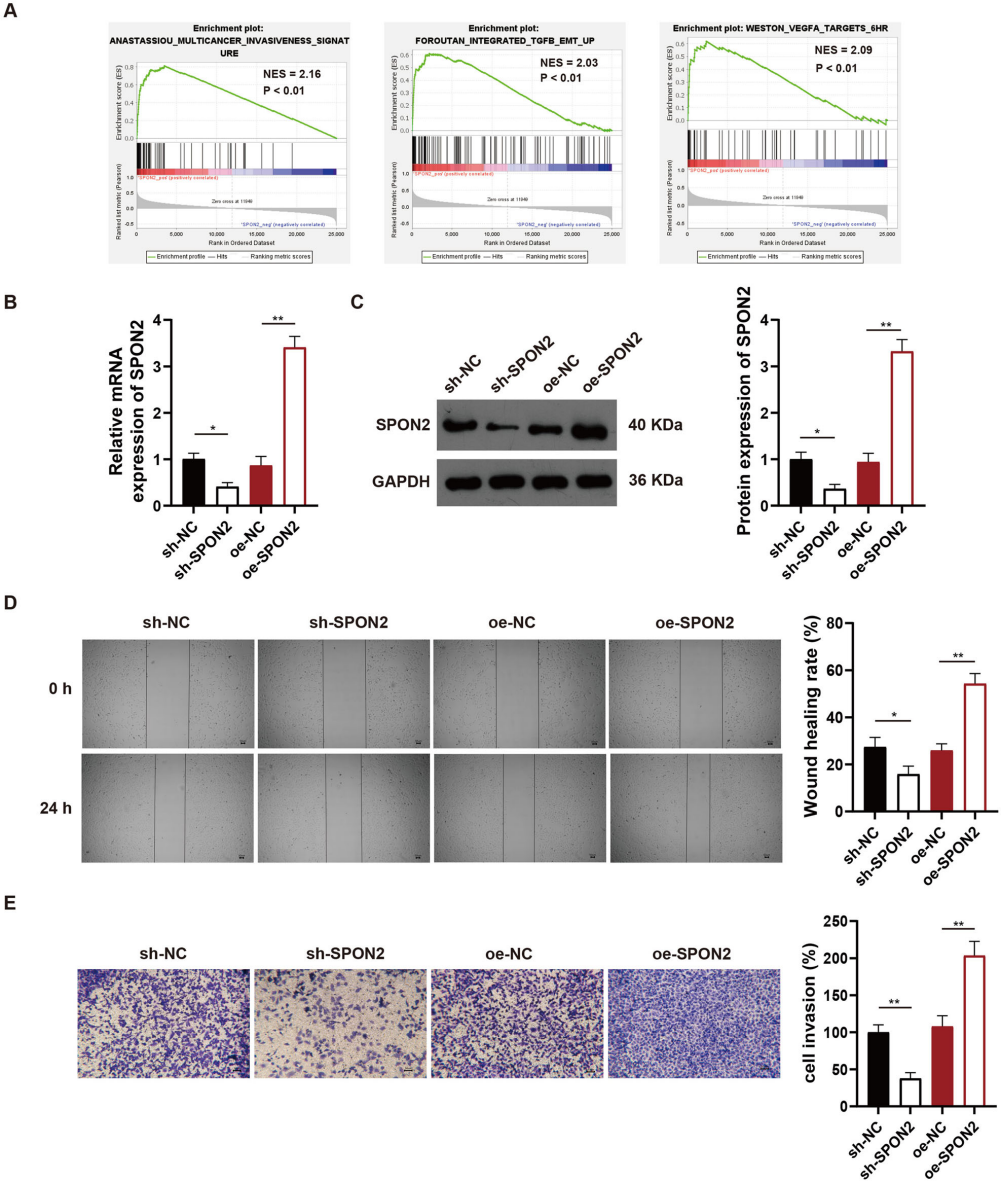
SPON2 promotes OS cell metastasis in vitro. **A** GSEA of transcriptomic data from GEO database (GSE21257) showing positive correlation between high SPON2 expression and enhanced OS metastasis. **B, C** Knockdown of SPON2 in MG63 cells using shRNA lentivirus (sh-SPON2) was confirmed by RT-qPCR and western blotting. **D, E** Wound healing and transwell invasion assays showing that SPON2 knockdown significantly suppressed OS cell migration and invasion. Conversely, overexpression of SPON2 increased migration and invasion capabilities. Scale bar: 50 μm. The student’s t-test (**B–E**) was utilized to ascertain group differences. The values are shown as the mean ± SD of three independent experiments. *p < 0.05, **p < 0.01.

To investigate the potential roles of SPON2 in OS, we first performed gene set enrichment analysis (GSEA) of transcriptomic data from the GEO database (GSE21257). The SPON2 expression level correlated positively with OS development. In addition, we found that high SPON2 expression was associated with enhanced OS metastasis ability (Fig. 2A), suggesting that SPON2 was a crucial factor promoting OS development and metastasis. To further validate these findings, we used lentiviral vectors to either reduce its expression using shRNA (sh- SPON2) or increase it through overexpression (oe-SPON2) (Fig. 2B, C), with sh-NC (non-targeting control) and oe-NC (empty vector control) as corresponding negative controls. We found that SPON2 knockdown significantly suppressed OS cell migration and invasion compared to the sh-NC control. Conversely, SPON2 overexpression markedly increased these metastatic capacities compared to the oe-NC control, as observed in the wound healing and transwell invasion assays (Fig. 2D, E). Collectively, these findings suggested that the upregulation of SPON2 contributes to OS cell migration and invasion in vitro.

**Fig. 3:**
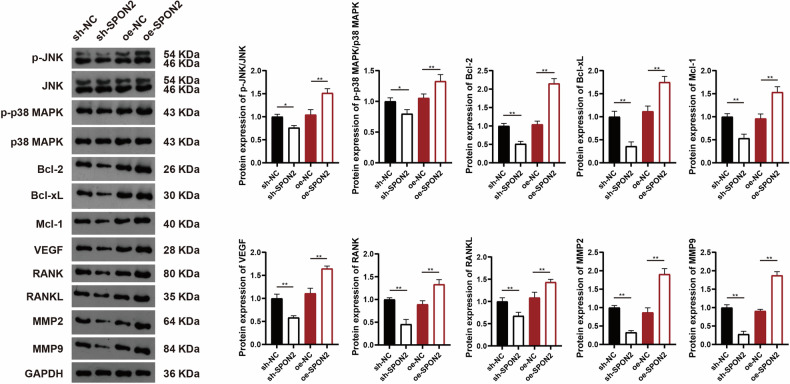
SPON2 regulates multiple signaling pathways to promote OS metastasis. Western blotting analysis of SPON2 knockdown and overexpression in MG63 cells showing the effects on p-JNK, p-p38 MAPK, Bcl-2, Bcl-xL, Mcl-1, VEGF, RANK, RANKL, MMP2, and MMP9 expression levels. GAPDH was used as the internal loading control in all immunoblot assays. Knockdown of SPON2 inhibits, while overexpression of SPON2 enhances these signaling pathways, indicating SPON2’s role in promoting tumor invasion and metastasis. Student’s t-test was employed for the study of differences between two groups. All data are presented as mean ± SD (*n* = 3 biological replicates). *p < 0.05, **p < 0.01.

Next, to investigate the mechanisms by which SPON2 influences OS cell migration, we further explored SPON2-regulated signaling pathways in OS. Notably, the western blotting demonstrated that SPON2 knockdown markedly inhibited the p-JNK, p-p38 MAPK, Bcl-2, Bcl-xL, Mcl-1, VEGF, RANK, RANKL, MMP2, and MMP9 expression levels compared to the sh-NC group. In contrast, SPON2 overexpression significantly increased the levels of these proteins relative to the oe-NC group (Fig. 3). Taken together, these findings suggested that SPON2 may promote tumor invasion and metastasis by activating the p38 MAPK pathway, anti-apoptotic signaling, angiogenesis pathway, RANK/RANKL signaling, and extracellular matrix degradation pathway.

### SPON2 promotes tumor proliferation, angiogenesis, and EMT in OS through NF-κB/VEGF signaling axis activation

**Fig. 4:**
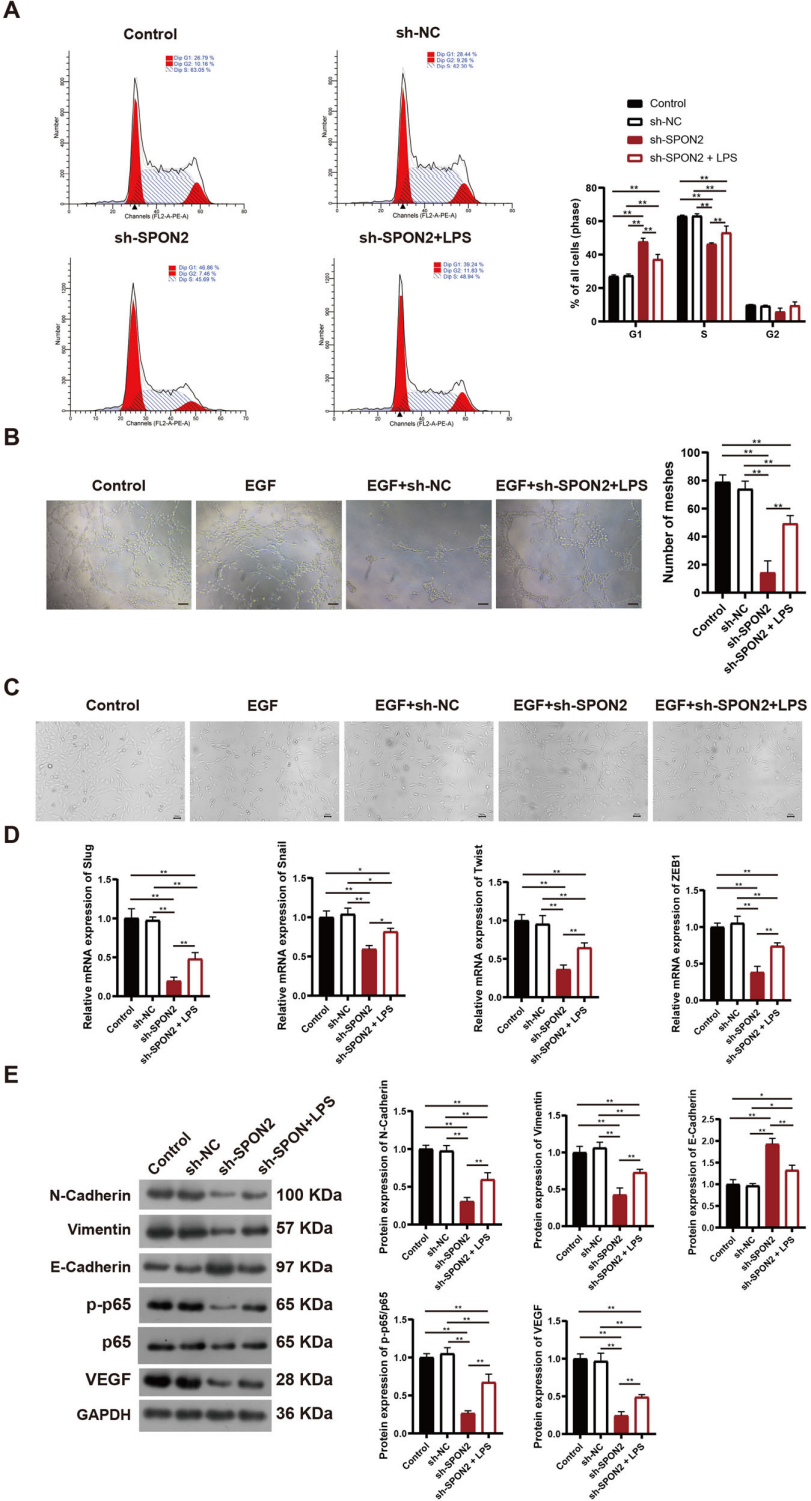
SPON2 promotes tumor cell proliferation, angiogenesis, and EMT in OS via NF-κB/VEGF signaling axis in vitro. **A** Flow cytometry analysis shows that SPON2 knockdown induces G1/G0 cell cycle arrest and reduces the proportion of S-phase cells in MG63 cells. **B** Tube formation assay in HUVECs reveals that SPON2 knockdown significantly inhibits angiogenesis, while treatment with 1 μg/mL LPS partially restores tube-forming capacity. Scale bar = 50 μm. **C** EGF treatment induces spindle-shaped morphology in MG63 cells, which is indicative of EMT. This change is blocked by SPON2 knockdown, while LPS treatment mitigates the effect, promoting EMT-like morphology. Scale bar = 50 μm. **D** RT-qPCR analysis shows that SPON2 knockdown reduces the expression of EMT-related transcription factors (Slug, Snail, Twist, and ZEB1). **E** Western blot analysis confirms that SPON2 knockdown decreases mesenchymal markers (N-cadherin and Vimentin) and increases the epithelial marker E-cadherin. LPS treatment reverses these changes and restores NF-κB p65 phosphorylation and VEGF expression. GAPDH was used as the internal control for all protein expression analyses. Data are presented as mean ± SD (*n* = 3 biological replicates). Statistical significance was determined using one-way ANOVA followed by Tukey’s post hoc test. *p < 0.05, **p < 0.01.

Although SPON2 has been reported to promote tumor proliferation and angiogenesis in various cancers [20, 27], its role in OS remains unexplored. To address this, we conducted flow cytometry and tube formation assays in OS cells. Flow cytometry revealed that SPON2 downregulation induced significant G1/G0 phase arrest and reduced the proportion of cells in the S phase in OS cells (Fig. 4A). Additionally, angiogenesis analysis using human umbilical vein endothelial cells (HUVECs) demonstrated that SPON2 knockdown significantly impaired tube formation, while treatment with 1 µg/mL LPS significantly improved tube-forming capacity compared to the knockdown group (*p* < 0.01) (Fig. 4B). The LPS concentration used in this study (1 µg/mL) has been widely used to activate NF-κB signaling in cancer cells without inducing cytotoxicity, providing a rationale for its inclusion in the rescue experiments [28]. These results indicate that SPON2 plays a crucial role in regulating tumor proliferation and angiogenesis in OS.

EMT is a critical process wherein epithelial cells lose their polarity and acquire mesenchymal characteristics, which contributes to tumor cell invasion and migration [29]. EMT can be triggered by a wide range of factors, including transforming growth factor beta (TGF-β), Wnt family proteins (WNTs), interleukin-6 (IL6), Notch signaling, epidermal growth factor (EGF), hepatocyte growth factor (HGF), fibroblast growth factor (FGF), and hypoxia-inducible factor (HIF) [30]. For example, EGF is known to trigger EMT by promoting E-cadherin endocytosis and upregulating ZEB1, a transcriptional repressor of E-cadherin and other targets [31]. LPS is another well-established inducer of EMT in epithelial cells [32]. In this study, we focused on EGF-induced changes in cell morphology and investigated whether SPON2 knockdown could inhibit EMT in OS cells. OS cells were transfected with a recombinant lentivirus carrying sh-SPON2 and subsequently treated with 50 ng/mL EGF and/or 1 µg/mL LPS. Figure 4C demonstrated that EGF treatment induced spindle-shaped, fibroblast-like morphology characteristic of EMT. However, SPON2 knockdown via sh-SPON2 lentivirus significantly inhibited these morphological changes, preserving the polygonal epithelial cell morphology. In contrast, treatment with 1 µg/mL LPS mitigated the morphological changes induced by SPON2 knockdown and promoted a spindle-like appearance comparable to that of the sh-NC group. To investigate the role of SPON2 in EMT, the expression levels of EMT-related transcription factors Slug, Snail, Twist, and ZEB1 were analyzed using reverse transcription-quantitative polymerase chain reaction (RT-qPCR), along with key EMT markers, including the epithelial marker E-cadherin and the mesenchymal markers N-cadherin and Vimentin. RT-qPCR results showed that SPON2 knockdown significantly downregulated the expression of Slug, Snail, Twist, and ZEB1 (Fig. 4D). Western blotting confirmed that SPON2 knockdown significantly reduced the expression of mesenchymal markers N-cadherin and Vimentin (*p* < 0.01), while markedly increasing E-cadherin levels (*p* < 0.01). Following treatment with 1 µg/mL LPS, these EMT-associated changes were significantly reversed compared with the sh-SPON2 group (*p* < 0.01), suggesting that SPON2 regulates EMT in OS cells in a manner that is sensitive to pro-inflammatory signaling (Fig. 4E).

The NF-κB signaling pathway is critical in promoting tumor growth, invasion, and metastasis by regulating angiogenesis, inflammation, and the tumor microenvironment. Previous studies have shown that SPON2 activates NF-κB signaling to facilitate EMT and enhance tumor progression [21, 33]. We next examined the impact of SPON2 on NF-κB signaling pathway. The results revealed that SPON2 knockdown in OS cells significantly reduced the expression of phosphorylated NF-κB p65 (p-p65) and a decreased p-p65 to total p65 ratio (*p* < 0.01), indicating suppressed NF-κB activation. Treatment with 1 µg/mL LPS significantly restored these levels compared to the knockdown group (*p* < 0.01), supporting the role of SPON2 in modulating NF-κB signaling in OS cells (Fig. 4E). Angiogenesis, which is crucial for tumor proliferation, invasion, and metastasis, supplies oxygen and nutrients to the tumor, facilitates cell dissemination, and creates a hypoxic, immune-suppressive microenvironment [34]. Tumor angiogenesis is regulated by the production of angiogenic factors, including members of the vascular endothelial growth factor (VEGF) family. Although previous studies have shown that the NF-κB pathway regulates VEGF expression [21], it remains unclear whether SPON2 is involved in this process in OS. To address this, we conducted further investigation. Western blotting confirmed that VEGF expression was significantly decreased in SPON2-knockdown OS cells (*p* < 0.01). Treatment with LPS significantly increased VEGF levels compared to the knockdown group (*p* < 0.01), though not to control levels (Fig. 4E). Taken together, these results indicate that SPON2 drives OS cell proliferation, angiogenesis, and EMT through the NF-κB/VEGF signaling axis, highlighting its potential as a therapeutic target in OS.

### SPON2 promotes M2 macrophage polarization by enhancing the secretion of IL10, CCL2, and CSF1

**Fig. 5:**
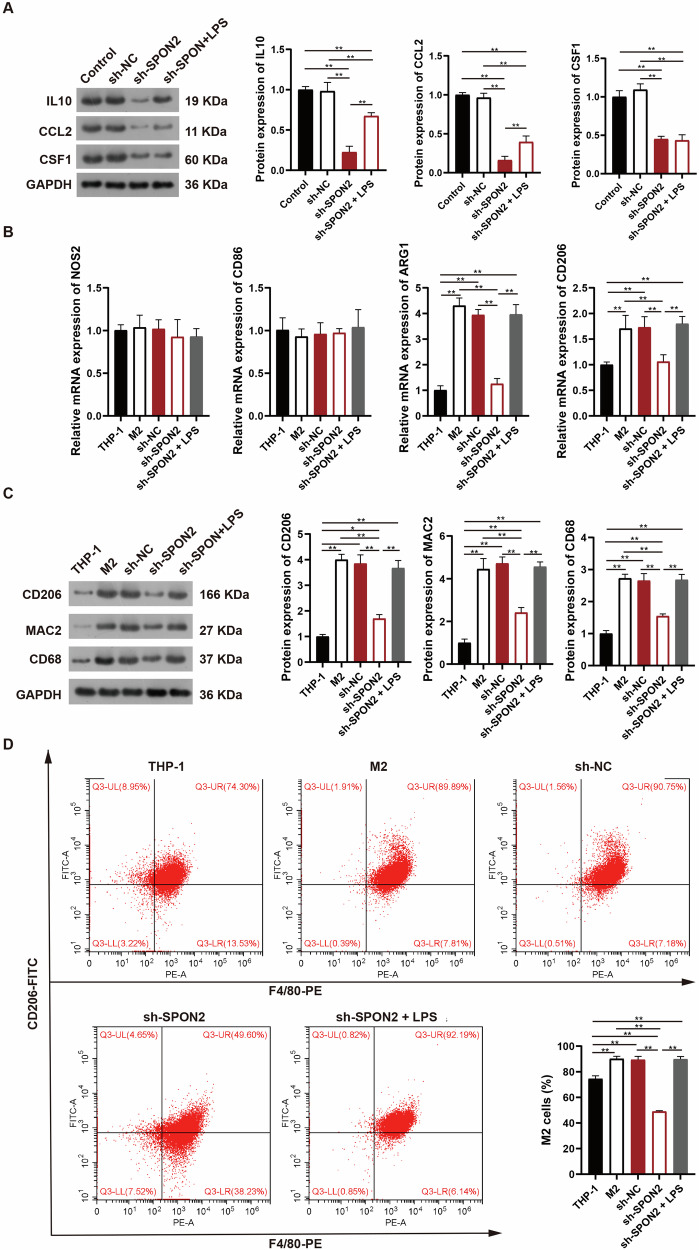
SPON2 regulates cytokine secretion and promotes M2 macrophage polarization in vitro. **A** Western blotting shows that SPON2 knockdown in MG63 cells reduces IL10, CCL2, and CSF1 secretion. LPS treatment restores IL10 and CCL2, but not CSF1. **B** RT-qPCR shows decreased expression of M2 markers (ARG1 and CD206) in THP-1 cells co-cultured with SPON2-knockdown MG63 cells. LPS treatment reverses these changes. **C** Western blotting reveals reduced levels of CD68, MAC-2, and CD206 in macrophages co-cultured with SPON2-knockdown OS cells, with LPS restoring expression. **D** Flow cytometry confirms that the proportion of F4/80⁺CD206⁺ cells is reduced in THP-1-derived macrophages co-cultured with SPON2-knockdown OS cells, but is partially restored following LPS treatment. Data are shown as mean ± SD (*n* = 3 biological replicates). One-way ANOVA with Tukey’s post-hoc test was used for statistical analysis. *p < 0.05, **p < 0.01.

Cytokines and chemokines secreted by tumor cells, such as IL10, CCL2, and CSF1, are key drivers of TAM plasticity within the pro-tumor microenvironment [35]. Previous studies have shown that SPON2 can enhance the secretion of these cytokines, thereby facilitating the polarization of human monocytic leukemia cells (THP-1) toward an M2 phenotype [25]. However, whether SPON2 exerts a similar effect in OS has not been thoroughly investigated. To address this, we first assessed the impact of SPON2 on cytokine secretion in OS cells. Western blotting results revealed that SPON2 knockdown significantly decreased IL10, CCL2, and CSF1 expression compared to their respective controls (*p* < 0.01). Following LPS stimulation, IL10 and CCL2 levels were significantly restored compared to the knockdown group (*p* < 0.01), while CSF1 expression remained unchanged (Fig. 5A). These findings suggest that SPON2 is involved in modulating cytokine secretion, which may contribute to macrophage polarization in OS.

We next performed transwell-based co-culture experiments to further evaluate the effect of SPON2 on macrophage polarization. MG63 OS cells (with or without SPON2 knockdown) were seeded in the lower chamber, and THP-1 cells were placed in the upper chamber. After co-culture, THP-1-derived macrophages were analyzed for M1/M2 polarization markers using RT-qPCR, western blotting, and flow cytometry. RT-qPCR analysis showed that SPON2 knockdown had no effect on the M1 markers NOS2 (iNOS) and CD86, whereas it significantly reduced the M2 markers ARG1 and CD206 compared to the M2 and sh-NC groups (*p* < 0.01, Fig. 5B). Notably, LPS treatment significantly increased CD206 and ARG1 expression levels relative to the sh-SPON2 group (*p* < 0.01), restoring them to levels comparable to those observed in the M2 and sh-NC groups (Fig. 5B). These findings were further supported at the protein level. Western blotting revealed that the expression of macrophage markers CD68 and MAC2 (Galectin-3), as well as the M2 marker CD206, was significantly reduced in THP-1 cells co-cultured with SPON2-knockdown OS cells, compared to the M2 and sh-NC controls (Fig. 5C). LPS treatment restored these markers to levels comparable to those in the M2 and sh-NC groups (Fig. 5C). Similarly, flow cytometry analysis confirmed that the percentage of F4/80⁺CD206⁺ macrophages was significantly decreased following SPON2 knockdown and recovered upon LPS treatment (Fig. 5D). The trends observed across RT-qPCR, western blot, and flow cytometry were consistent. Collectively, these findings suggest that SPON2 promotes M2 polarization of macrophages in vitro, at least in part, through the regulation of cytokine secretion and inflammatory signaling.

### SPON2 promotes macrophage M2 polarization and tumor invasion via NF-κB/VEGF signaling axis in vivo

**Fig. 6:**
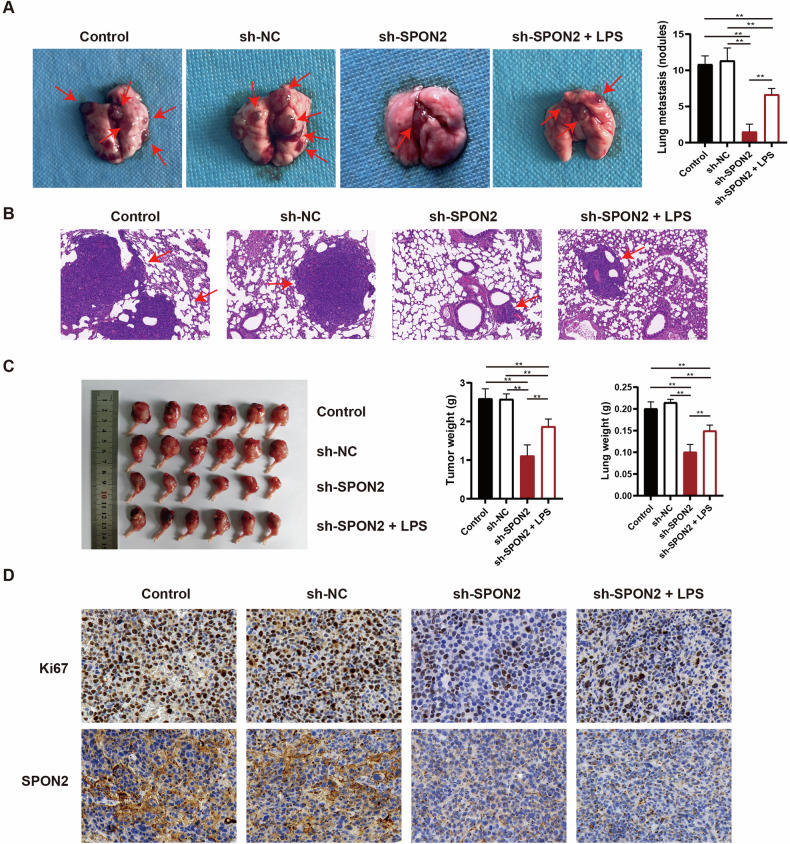
SPON2 knockdown suppresses tumor growth and metastasis in vivo. **A** Lung metastatic nodules are fewer in mice injected with sh-SPON2 K7M2 cells. Arrowheads indicate the metastatic nodules. **B** H&E staining of lung tissue confirms the reduction in metastatic lesions in the sh-SPON2 group. Scale bar = 100 μm. **C** Tumor and lung weights are significantly reduced in the SPON2 knockdown group compared to controls. **D** Ki67 immunostaining shows reduced tumor cell proliferation in the sh-SPON2 group. Scale bar = 50 μm. LPS pretreatment of SPON2-knockdown cells results in moderate increases in lung metastases, tumor and lung weights, and Ki67 expression, attenuating the inhibitory effects. Data are presented as mean ± SD (*n* = 6 biologically independent samples). Statistical analysis is performed using one-way ANOVA followed by Tukey’s post-hoc test (**A–C**). **p < 0.01.

To examine the role of SPON2 in OS progression in vivo, highly metastatic murine OS cells (K7M2 WT) were stably infected with lentiviruses encoding either sh-NC or sh-SPON2. The cells were injected into the proximal tibia of BALB/C nude mice (*n* = 6 per group), and tumor size was assessed over five weeks. At the end of the experiment, the lungs and lower limbs were harvested for analysis. Mice injected with SPON2-knockdown cells developed fewer lung metastases (Fig. 6A), exhibited reduced metastatic lesions by haematoxylin and eosin (H&E) staining (Fig. 6B), and showed lower tumor and lung weights compared to the control and sh-NC groups (Fig. 6C). Ki67 staining also revealed decreased tumor cell proliferation in the sh-SPON2 group (Fig. 6D). When SPON2-knockdown cells were pretreated with LPS, these inhibitory effects were partially alleviated. LPS stimulation led to modest increases in lung metastatic nodules, tumor and lung weights, and Ki67 expression compared to the sh-SPON2 group (Fig. 6A–D).

**Fig. 7:**
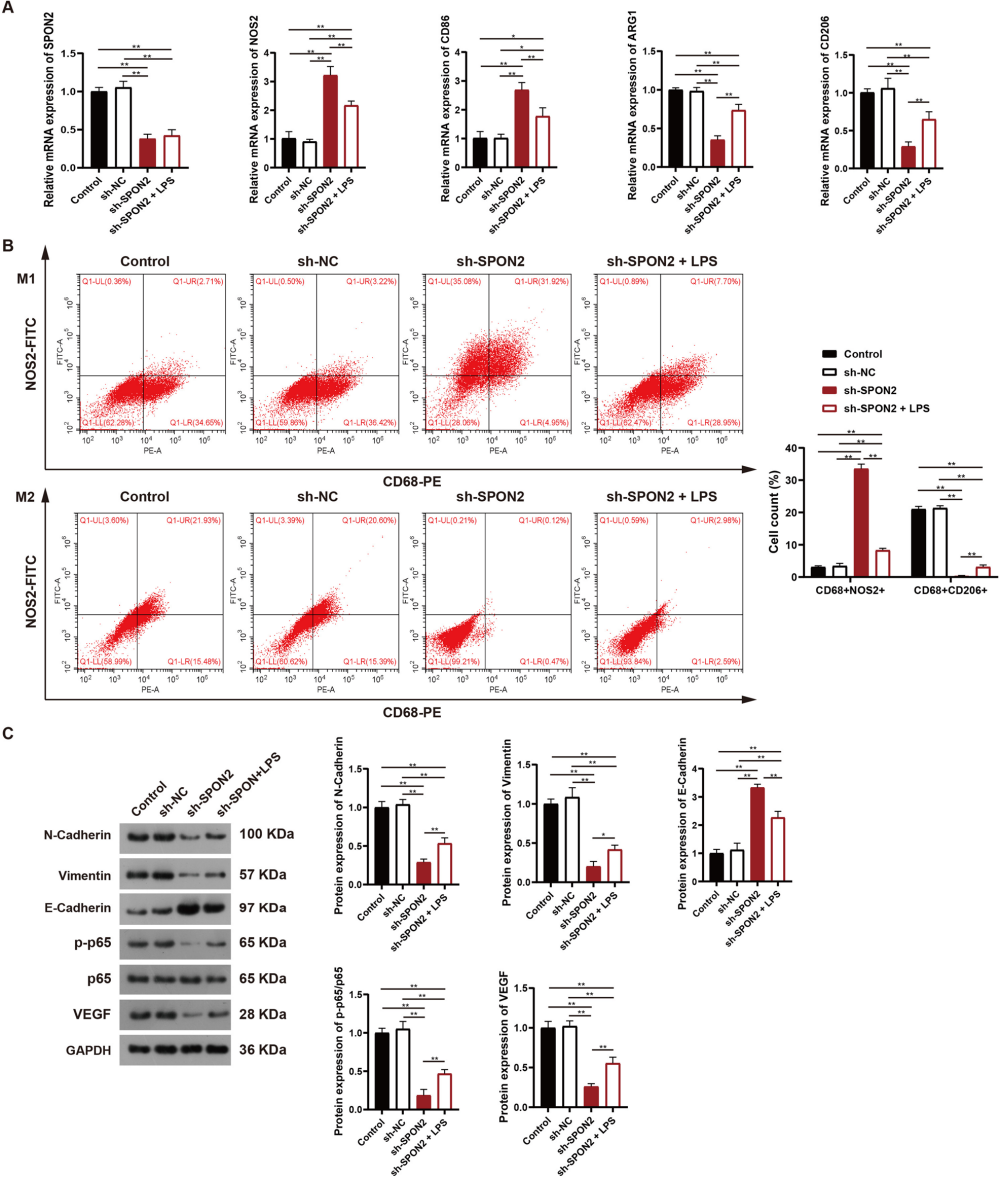
SPON2 knockdown regulates macrophage polarization and inhibits EMT via the NF-κB/VEGF signaling axis in vivo. **A** RT-qPCR analysis of tumor tissues from mice shows decreased expression of M2 markers (CD206 and ARG1) and increased expression of M1 markers (NOS2 and CD86) in the sh-SPON2 group. **B** Flow cytometry confirms lower levels of CD68 and CD206, and a higher proportion of CD68⁺NOS2⁺ macrophages following SPON2 knockdown. LPS treatment restores M2 markers and reduces M1 marker levels. **C** Western blotting shows that SPON2 knockdown increases E-cadherin and decreases N-cadherin, Vimentin, NF-κB p65, and VEGF in tumor tissues. These changes are attenuated by LPS treatment. Data are shown as mean ± SD (*n* = 6 biologically independent samples). One-way ANOVA followed by Tukey’s post-hoc test was used. *p < 0.05, **p < 0.01.

To explore the impact of SPON2 inhibition on macrophage polarization in vivo, tumor tissues were analyzed by RT-qPCR and flow cytometry. As expected, RT-qPCR confirmed successful SPON2 knockdown and showed reduced expression of M2 markers (CD206 and ARG1) along with elevated M1 markers (NOS2 and CD86) in the sh-SPON2 group (Fig. 7A). Flow cytometry results aligned with these findings, showing decreased levels of CD206 and CD68, and increased CD68⁺NOS2⁺ cells (Fig. 7B). Notably, LPS administration in SPON2-knockdown tumors attenuated these effects, partially restoring M2 marker expression and lowering M1 marker levels (Fig. 7B). Together, these results indicate that SPON2 promotes M2 macrophage polarization in vivo, and LPS can partially reverse the polarization defect induced by SPON2 knockdown.

To further investigate the mechanism by which SPON2 influences macrophage behavior and tumor progression in vivo, we examined whether it affects EMT through NF-κB/VEGF signaling axis. Western blot analysis of tumor tissues from nude mice showed that SPON2 knockdown significantly increased E-cadherin levels while reducing the expression of N-cadherin, Vimentin, NF-κB p65, and VEGF (Fig. 7C), indicating a suppression of EMT. Interestingly, these changes were partly reversed by LPS treatment, supporting the hypothesis that SPON2 promotes EMT and macrophage M2 polarization in vivo via activation of the NF-κB/VEGF pathway (Fig. 7C).

## Discussion

OS is a highly aggressive bone tumor with a tendency to metastasize to the lungs, often resulting in poor prognosis despite advances in surgical resection and chemotherapy [1]. The molecular mechanisms driving OS progression, particularly those related to proliferation and metastasis, remain insufficiently understood [36]. In this study, we identify SPON2 as a key modulator of OS progression, highlighting its role in enhancing tumor cell proliferation, facilitating metastasis, promoting EMT, and modulating macrophage polarization. We further delineate the involvement of the NF-κB/VEGF signaling pathway in SPON2-mediated regulation of tumor invasion and angiogenesis. These findings provide new insights into the molecular landscape of OS progression and suggest that targeting SPON2 may offer a promising therapeutic strategy.

In this study, we found that SPON2 is significantly upregulated in both OS cell lines and primary OS tumors samples, and its elevated expression is correlated with poorer prognosis in OS patients. These findings highlight SPON2 as a potential prognostic biomarker in OS. Notably, the upregulation of SPON2 has also been observed in multiple other solid tumors, where it similarly correlates with poor prognosis, suggesting its potential as a universal biomarker and therapeutic target. This is consistent with prior studies reporting that elevated SPON2 expression is linked to tumor aggressiveness and poor prognosis in other cancer types, such as breast, gastric, and colorectal cancers [19, 20, 25, 27].

Our findings suggest that SPON2 contributes to OS progression by engaging multiple signaling pathways involved in cell migration and invasion. In vitro assays showed that SPON2 knockdown reduced, while its overexpression enhanced, the motility and invasiveness of OS cells. Mechanistically, changes in SPON2 expression were accompanied by altered activity of several key molecules. SPON2 silencing led to decreased phosphorylation of p38 MAPK and JNK, reduced levels of anti-apoptotic proteins such as Bcl-2, Bcl-xL, and Mcl-1, and downregulation of VEGF, RANK, RANKL, and the matrix metalloproteinases MMP2 and MMP9. These factors are known to promote tumor cell survival, vascular development, bone resorption, and matrix remodeling, all of which contribute to the metastatic potential of OS. The observation that SPON2 upregulation enhances multiple of these pathways suggests that it may act upstream in coordinating a pro-metastatic signaling network. This is in line with earlier studies showing that MAPK signaling and MMP activity support OS invasion and progression [37, 38].

We further explored how SPON2 contributes to the invasive behavior of OS cells and found clear evidence of its involvement in EMT. SPON2 knockdown led to increased E-cadherin expression and decreased levels of N-cadherin and Vimentin, indicating suppression of the mesenchymal phenotype. These molecular changes were accompanied by reduced expression of EMT-related transcription factors, including Slug, Snail, Twist, and ZEB1. Since EMT is a key step in tumor metastasis, these results suggest that SPON2 enhances OS cell plasticity and invasiveness through EMT induction. The observed decrease in p-p65 and VEGF following SPON2 knockdown implies that SPON2 may act upstream of NF-κB signaling and VEGF-mediated angiogenesis. Given that NF-κB is known to promote EMT and drive VEGF-mediated angiogenesis, it is likely that SPON2 exerts its effects at least in part through activation of NF-κB signaling. This notion is supported by previous studies in other cancer types showing NF-κB-dependent EMT progression and VEGF upregulation [39, 40]. Our findings thus place SPON2 within a signaling context that connects inflammation, EMT, and angiogenesis, reinforcing its role in promoting an aggressive tumor phenotype.

Our study reveals a previously unrecognized role of SPON2 in shaping the tumor immune microenvironment by promoting macrophage polarization in OS. We found that SPON2 enhances the secretion of cytokines such as IL10, CCL2, and CSF1, which drive polarization toward the M2 phenotype and contribute to a pro-tumorigenic environment. This aligns with prior reports in other malignancies, where SPON2 has been shown to promote cytokine production and support tumor-promoting immune responses [19, 25]. Interestingly, LPS stimulation restored IL10 and CCL2 expression but had no effect on CSF1 levels in SPON2-knockdown cells. This suggests that SPON2 may regulate CSF1 through pathways distinct from the LPS-activated NF-κB signaling, highlighting a degree of selectivity in its modulation of cytokine expression. However, to our knowledge, this is the first study demonstrating that SPON2 regulates macrophage polarization in OS. These findings are particularly relevant given the well-established role of M2 macrophages in enhancing immune suppression, angiogenesis, and matrix remodeling. By driving macrophage polarization toward the M2 subtype, SPON2 may contribute to immune evasion and therapeutic resistance in OS. Targeting this immunomodulatory axis could provide a novel strategy to reshape the tumor microenvironment and improve treatment outcomes.

The partial restoration of tumor-promoting features by LPS in SPON2-knockdown models supports the involvement of NF-κB/VEGF signaling in SPON2-related effects. As an established activator of NF-κB, LPS was able to recover M2 macrophage marker expression, EMT-related protein levels, and metastatic behavior to some extent. These observations suggest that inflammatory signals can, at least partially, substitute for SPON2 function, pointing to a connection between SPON2 activity and broader immune or inflammatory pathways in the tumor microenvironment. The responsiveness to LPS also raises the possibility that targeting innate immune signaling could offer an alternative way to influence macrophage polarization and EMT in OS. Further work is needed to understand how endogenous inflammatory factors interact with SPON2-regulated pathways in shaping tumor progression.

Given SPON2’s dual role in promoting both tumor progression and immune modulation, it presents a compelling target for therapeutic intervention. Several therapeutic approaches could be explored, including the development of monoclonal antibodies or small molecules that target SPON2 or its signaling intermediates. Inhibiting NF-κB signaling with specific inhibitors such as bortezomib, or targeting VEGF signaling with monoclonal antibodies like bevacizumab, could also prove effective in OS. Additionally, strategies aimed at reprogramming M2 macrophages to an M1 phenotype or inhibiting their polarization altogether may offer a novel approach for disrupting the immunosuppressive TME in OS. However, before SPON2-targeted therapies can be translated into clinical practice, further studies are needed to assess the feasibility and safety of such approaches. Preclinical and clinical trials will be essential for evaluating the therapeutic potential of SPON2 inhibition in OS and other cancers with similar TME dynamics.

While our study provides significant insights into the role of SPON2 in OS progression, there are several limitations. First, while we demonstrate the importance of SPON2 in OS cell lines and xenograft models, the findings should be validated in larger, more heterogeneous patient cohorts to confirm their clinical relevance. Second, the precise molecular mechanisms by which SPON2 regulates macrophage polarization and EMT in OS remain to be fully elucidated. Future studies should explore the signaling cascades involved in SPON2-mediated polarization and investigate how these pathways intersect with other immune and stromal components in the TME. Furthermore, while SPON2 has been shown to play a significant role in OS, its function in other cancers, particularly those with similar immune landscape characteristics, warrants further investigation. Expanding the scope of SPON2 research to include a broader range of tumor types could provide valuable insights into its generalizability as a therapeutic target.

**Fig. 8:**
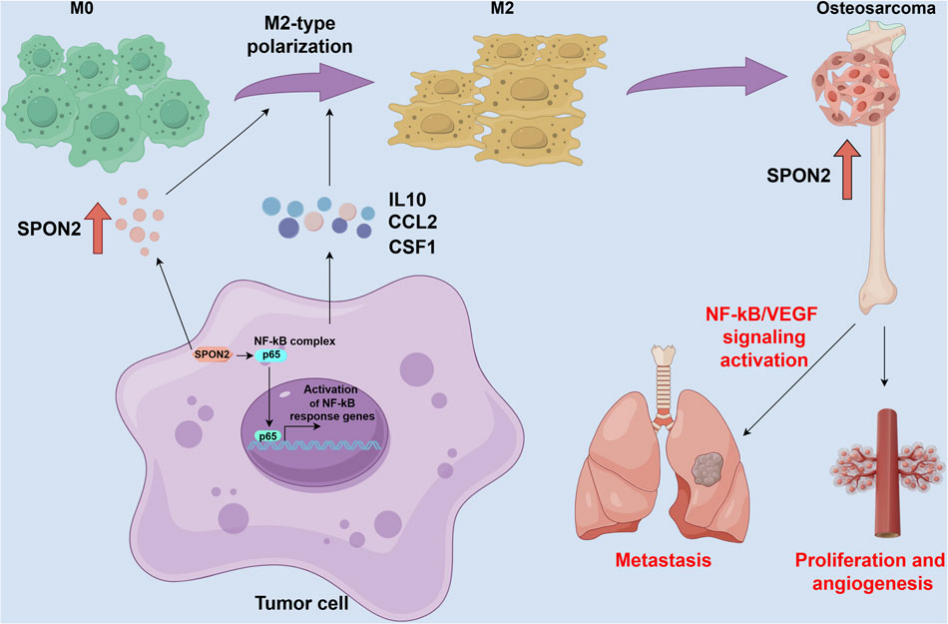
Proposed Working Model. SPON2 is significantly upregulated in OS, driving tumor proliferation and metastasis. Mechanistically, SPON2 promotes M2 macrophage polarization by activating the NF-κB/VEGF signaling axis and modulating cytokine secretion, including IL10, CCL2, and CSF1, thereby fostering an immunosuppressive tumor microenvironment that facilitates OS progression.

In summary, our study identifies SPON2 as a key contributor to OS progression by facilitating tumor cell proliferation, EMT, angiogenesis, and M2 macrophage polarization through the NF-κB/VEGF signaling axis. Moreover, SPON2 enhances the secretion of IL10, CCL2, and CSF1, supporting an immunosuppressive microenvironment that promotes OS cell proliferation, invasion, and metastasis (Fig. 8). These findings highlight SPON2 as both a potential biomarker and a therapeutic target in OS, with broader relevance to other tumors sharing similar inflammatory and stromal features.

## Materials and Methods

### Cell culture

The cell lines used in this study included MG63, 143B, hFOB1.19, THP-1, K7M2 WT, and HUVECs, all obtained from certified suppliers and cultured under standard conditions. Detailed information on cell sources and culture methods is provided in the Supplementary Methods.

### RT-qPCR

Total RNA was extracted and reverse transcribed into cDNA, followed by quantitative PCR using SYBR Green chemistry with GAPDH as the internal control. Detailed procedures and primer sequences are available in the Supplementary Methods and Supplementary Table 1.

### Western blotting

Cells were lysed and proteins were extracted for separation by SDS-PAGE, followed by transfer to PVDF membranes and detection using specific antibodies. Detailed western blotting procedures and antibody information are provided in the Supplementary Methods and Supplementary Table 2.

### Lentiviral vectors construction and transfection

Lentiviral vectors for SPON2 overexpression and knockdown were constructed and transfected according to standard protocols, with stable cells selected using puromycin. Detailed vector construction, sequences, and transfection procedures are provided in the Supplementary Methods.

### Wound healing, transwell, cell cycle, and tube formation assays

Wound healing, transwell, cell cycle, and tube formation assays were conducted to assess cell migration, proliferation, and angiogenesis under various experimental conditions. Detailed protocols for each assay are provided in the Supplementary Methods.

### Cell morphology, EMT induction, macrophage polarization, and co-culture assays

Unless otherwise stated, cells were treated with or without 1 µg/mL LPS (Sigma-Aldrich, L3024, USA) to evaluate its ability to reverse the effects of SPON2 knockdown. This concentration is widely used in in vitro studies to activate NF-κB signaling without causing cytotoxicity [28], and was applied to both OS cells and co-culture systems. Cell morphology, EMT induction, macrophage polarization, and co-culture assays were performed to investigate the role of SPON2 in EMT regulation and immune modulation in OS. Detailed experimental design, treatment conditions, and detection methods are provided in the Supplementary Methods.

### Animal works

The animal experiments were approved by the Animal Ethical Committee of the First Affiliated Hospital of Zhengzhou University, and were conducted in accordance with the Management and Use Guidelines of Laboratory Animals of NIH. Tumor xenograft assays were performed in BALB/C nude mice to assess the impact of SPON2 knockdown on tumor growth, metastasis, EMT, and macrophage polarization. Detailed experimental procedures and detection methods are provided in the Supplementary Methods.

### Flow cytometry and IHC analysis

Flow cytometry and IHC analysis were used to analyze macrophage phenotypes and the expression of Ki67 and SPON2 in tumor tissues. Detailed procedures, antibody information, and analysis methods are provided in the Supplementary Methods and Supplementary Table 2.

### GSEA and pan-cancer analysis

GSEA and pan-cancer analysis were conducted to explore the biological pathways and prognostic relevance associated with SPON2 expression. Detailed datasets, analytical methods, and statistical criteria are provided in the Supplementary Methods.

### Statistical analysis

All quantitative data are expressed as mean ± standard deviation (SD). The Student’s t-test, Wilcoxon rank-sum test, and one-way ANOVA with Tukey’s post hoc test were employed to compare continuous variables between groups. Survival data were illustrated using Kaplan–Meier curves, and survival differences were assessed by the log-rank test. Statistical analysis was conducted using GraphPad Prism (version 8). A *p*-value of less than 0.05 was employed to denote statistical significance.

## Supplementary information

Supplementary Methods

Supplementary Table 1

Supplementary Table 2

Original Western blot file

## Data Availability

The authors declare that all other data supporting the findings of this study are available within the article and its supplementary materials. Additional data are available from the corresponding author upon reasonable request.

## References

[1] Bielack SS, Kempf-Bielack B, Delling G, Exner GU, Flege S, Helmke K, et al. Prognostic factors in high-grade osteosarcoma of the extremities or trunk: an analysis of 1,702 patients treated on neoadjuvant cooperative osteosarcoma study group protocols. J Clin. Oncol. 2002;20:776–90. 11821461 10.1200/JCO.2002.20.3.776

[2] Bielack SS, Kempf-Bielack B, Branscheid D, Carrle D, Friedel G, Helmke K, et al. Second and subsequent recurrences of osteosarcoma: presentation, treatment, and outcomes of 249 consecutive cooperative osteosarcoma study group patients. J Clin. Oncol. 2009;27:557–65. 19075282 10.1200/JCO.2008.16.2305

[3] Meltzer PS, Helman LJ. New horizons in the treatment of Osteosarcoma. N Engl. J. Med. 2021;385:2066–76. 34818481 10.1056/NEJMra2103423

[4] Kager L, Zoubek A, Pötschger U, Kastner U, Flege S, Kempf-Bielack B, et al. Primary metastatic osteosarcoma: presentation and outcome of patients treated on neoadjuvant Cooperative Osteosarcoma Study Group protocols. J Clin. Oncol. 2003;21:2011–8. 12743156 10.1200/JCO.2003.08.132

[5] Li C, Xu X, Wei S, Jiang P, Xue L, Wang J. Tumor-associated macrophages: potential therapeutic strategies and future prospects in cancer. J Immunother Cancer. 2021;9:e001341. 10.1136/jitc-2020-001341 PMC8728363 33504575

[6] Zhou Y, Yang D, Yang Q, Lv X, Huang W, Zhou Z, et al. Single-cell RNA landscape of intratumoral heterogeneity and immunosuppressive microenvironment in advanced osteosarcoma. Nat Commun. 2020;11:6322. 33303760 10.1038/s41467-020-20059-6 PMC7730477

[7] Mantovani A, Marchesi F, Malesci A, Laghi L, Allavena P. Tumour-associated macrophages as treatment targets in oncology. Nat Rev. Clin. Oncol. 2017;14:399–416. 28117416 10.1038/nrclinonc.2016.217 PMC5480600

[8] Murray PJ, Wynn TA. Protective and pathogenic functions of macrophage subsets. Nat Rev. Immunol. 2011;11:723–37. 21997792 10.1038/nri3073 PMC3422549

[9] Cersosimo F, Lonardi S, Bernardini G, Telfer B, Mandelli GE, Santucci A, et al. Tumor-associated macrophages in osteosarcoma: from mechanisms to therapy. Int J Mol Sci. 2020;21:5207. 10.3390/ijms21155207 PMC7432207 32717819

[10] Huang Q, Liang X, Ren T, Huang Y, Zhang H, Yu Y, et al. The role of tumor-associated macrophages in osteosarcoma progression - therapeutic implications. Cell Oncol. 2021;44:525–39. 10.1007/s13402-021-00598-w PMC12980758 33788151

[11] Zhao Y, Zhang B, Zhang Q, Ma X, Feng H. Tumor-associated macrophages in osteosarcoma. J Zhejiang Univ. Sci. B. 2021;22:885–92. 34783219 10.1631/jzus.B2100029 PMC8593527

[12] Li Y, Cao C, Jia W, Yu L, Mo M, Wang Q, et al. Structure of the F-spondin domain of mindin, an integrin ligand and pattern recognition molecule. EMBO J. 2009;28:286–97. 19153605 10.1038/emboj.2008.288 PMC2637340

[13] Jia W, Li H, He YW. The extracellular matrix protein mindin serves as an integrin ligand and is critical for inflammatory cell recruitment. Blood. 2005;106:3854–9. 16105980 10.1182/blood-2005-04-1658 PMC1895097

[14] Feinstein Y, Klar A. The neuronal class 2 TSR proteins F-spondin and Mindin: a small family with divergent biological activities. Int J. Biochem Cell Biol. 2004;36:975–80. 15094111 10.1016/j.biocel.2004.01.002

[15] Li N, Liu S, Zhang Y, Yu L, Hu Y, Wu T, et al. Transcriptional activation of matricellular protein Spondin2 (SPON2) by BRG1 in vascular endothelial cells promotes macrophage Chemotaxis. Front Cell Dev. Biol. 2020;8:794. 32974343 10.3389/fcell.2020.00794 PMC7461951

[16] Liu YS, Wang LF, Cheng XS, Huo YN, Ouyang XM, Liang LY, et al. The pattern-recognition molecule mindin binds integrin Mac-1 to promote macrophage phagocytosis via Syk activation and NF-κB p65 translocation. J Cell Mol. Med. 2019;23:3402–16. 30869196 10.1111/jcmm.14236 PMC6484411

[17] Inai Y, Ueda K, Matsui IL, Tajiri M, Minakata S, Wada Y, et al. Role of C-mannosylation in the secretion of mindin. Biochim Biophys. Acta Gen. Subj. 2020;1864:129632. 32416197 10.1016/j.bbagen.2020.129632

[18] Jia W, Li H, He YW. Pattern recognition molecule mindin promotes intranasal clearance of influenza viruses. J Immunol. 2008;180:6255–61. 18424748 10.4049/jimmunol.180.9.6255

[19] Hu X, Su C, Wei J. Knockdown of SPON2 inhibits the growth of triple-negative breast cancer. Front Oncol. 2023;13:1141417. 36959811 10.3389/fonc.2023.1141417 PMC10029917

[20] Kang HG, Kim WJ, Noh MG, Chun KH, Kim SJ. SPON2 Is Upregulated through Notch Signaling Pathway and Promotes Tumor Progression in Gastric. Cancer. Cancers. 2020;12:1439. 10.3390/cancers12061439 PMC7352369 32492954

[21] Wu M, Kong D, Zhang Y. SPON2 promotes the bone metastasis of lung adenocarcinoma via activation of the NF-κB signaling pathway. Bone. 2023;167:116630. 36427776 10.1016/j.bone.2022.116630

[22] Yuan X, Bian T, Liu J, Ke H, Feng J, Zhang Q, et al. Spondin2 is a new prognostic biomarker for lung adenocarcinoma. Oncotarget. 2017;8:59324–32. 28938639 10.18632/oncotarget.19577 PMC5601735

[23] Lucarelli G, Rutigliano M, Bettocchi C, Palazzo S, Vavallo A, Galleggiante V, et al. Spondin-2, a secreted extracellular matrix protein, is a novel diagnostic biomarker for prostate cancer. J Urol. 2013;190:2271–7. 23665271 10.1016/j.juro.2013.05.004

[24] Feng Y, Hu Y, Mao Q, Guo Y, Liu Y, Xue W, et al. Upregulation of Spondin-2 protein expression correlates with poor prognosis in hepatocellular carcinoma. J Int Med Res. 2019;47:569–79. 30318967 10.1177/0300060518803232 PMC6381490

[25] Huang C, Ou R, Chen X, Zhang Y, Li J, Liang Y, et al. Tumor cell-derived SPON2 promotes M2-polarized tumor-associated macrophage infiltration and cancer progression by activating PYK2 in CRC. J Exp. Clin. Cancer Res. 2021;40:304. 34583750 10.1186/s13046-021-02108-0 PMC8477524

[26] Wang S, Yan Y, Cheng Z, Hu Y, Liu T. Sotetsuflavone suppresses invasion and metastasis in non-small-cell lung cancer A549 cells by reversing EMT via the TNF-α/NF-κB and PI3K/AKT signaling pathway. Cell Death Discov. 2018;4:26. 10.1038/s41420-018-0026-9 PMC5841291 29531823

[27] Pan R, Yu Y, Zhu H, Zhang W, Qin Y, Ye L, et al. RSPO2 promotes progression of ovarian cancer through dual receptor-mediated FAK/Src signaling activation. iScience. 2022;25:105184. 36217544 10.1016/j.isci.2022.105184 PMC9547309

[28] Guo L, Zhang Y, Wei R, Wang C, Feng M. Lipopolysaccharide-anchored macrophages hijack tumor microtube networks for selective drug transport and augmentation of antitumor effects in orthotopic lung cancer. Theranostics. 2019;9:6936–48. 31660078 10.7150/thno.37380 PMC6815965

[29] Dongre A, Weinberg RA. New insights into the mechanisms of epithelial-mesenchymal transition and implications for cancer. Nat Rev. Mol. Cell Biol. 2019;20:69–84. 30459476 10.1038/s41580-018-0080-4

[30] Thiery JP, Acloque H, Huang RY, Nieto MA. Epithelial-mesenchymal transitions in development and disease. Cell. 2009;139:871–90. 19945376 10.1016/j.cell.2009.11.007

[31] Lu Z, Ghosh S, Wang Z, Hunter T. Downregulation of caveolin-1 function by EGF leads to the loss of E-cadherin, increased transcriptional activity of beta-catenin, and enhanced tumor cell invasion. Cancer Cell. 2003;4:499–515. 14706341 10.1016/s1535-6108(03)00304-0

[32] Jing YY, Han ZP, Sun K, Zhang SS, Hou J, Liu Y, et al. Toll-like receptor 4 signaling promotes epithelial-mesenchymal transition in human hepatocellular carcinoma induced by lipopolysaccharide. BMC Med. 2012;10:98. 22938142 10.1186/1741-7015-10-98 PMC3482562

[33] Termini CM, Pang A, Fang T, Roos M, Chang VY, Zhang Y, et al. Neuropilin 1 regulates bone marrow vascular regeneration and hematopoietic reconstitution. Nat Commun. 2021;12:6990. 34848712 10.1038/s41467-021-27263-y PMC8635308

[34] Li X, Chen Y, Lan R, Liu P, Xiong K, Teng H, et al. Transmembrane mucins in lung adenocarcinoma: understanding of current molecular mechanisms and clinical applications. Cell Death Discov. 2025;11:163. 40210618 10.1038/s41420-025-02455-3 PMC11985918

[35] Suzuki J, Aokage K, Neri S, Sakai T, Hashimoto H, Su Y, et al. Relationship between podoplanin-expressing cancer-associated fibroblasts and the immune microenvironment of early lung squamous cell carcinoma. Lung Cancer. 2021;153:1–10. 33429158 10.1016/j.lungcan.2020.12.020

[36] Espejo Valle-Inclan J, De Noon S, Trevers K, Elrick H, van Belzen I, Zumalave S, et al. Ongoing chromothripsis underpins osteosarcoma genome complexity and clonal evolution. Cell. 2025;188:352–70.e322. 39814020 10.1016/j.cell.2024.12.005

[37] Zhong B, Shi D, Wu F, Wang S, Hu H, Cheng C, et al. Dynasore suppresses cell proliferation, migration, and invasion and enhances the antitumor capacity of cisplatin via STAT3 pathway in osteosarcoma. Cell Death Dis. 2019;10:687. 31534119 10.1038/s41419-019-1917-2 PMC6751204

[38] Liu JF, Chen PC, Chang TM, Hou CH. Monocyte Chemoattractant Protein-1 promotes cancer cell migration via c-Raf/MAPK/AP-1 pathway and MMP-9 production in osteosarcoma. J Exp. Clin. Cancer Res. 2020;39:254. 33228783 10.1186/s13046-020-01756-y PMC7684958

[39] Chen X, Weng Y, Li Y, Fu W, Huang Z, Pan Y, et al. Upregulation of PNCK Promotes Metastasis and Angiogenesis via Activating NF-κB/VEGF Pathway in Nasopharyngeal Carcinoma. J Oncol. 2022;2022:8541582. 35535310 10.1155/2022/8541582 PMC9078829

[40] Chen T, Ruan Y, Ji L, Cai J, Tong M, Xue Y, et al. S100A6 drives lymphatic metastasis of liver cancer via activation of the RAGE/NF-kB/VEGF-D pathway. Cancer Lett. 2024;587:216709. 38350547 10.1016/j.canlet.2024.216709

