# Supplementary material for: SPON2 facilitates osteosarcoma development by inducing M2 macrophage polarization through activation of the NF-κB/VEGF signaling axis
Source: Cell Death Discov. 2025 Jul 29;11:352. doi: 10.1038/s41420-025-02626-2 (PMC12307574; doi:10.1038/s41420-025-02626-2)
Supplement: Supplementary file 1 — Supplementary Methods [file 41420_2025_2626_MOESM1_ESM.docx]

**Supplementary Methods**

**SPON2 facilitates osteosarcoma development by inducing M2 macrophage polarization through activation of the NF-κB/VEGF signaling axis**

Xinchang Lu^1,†^ Xueping Zhang^2,†^, Fengzhen Zhang^3^, Wenhao Wang^3^, Ruijie Liu^3^, Yubao Hou^3^, Weiye Shi^3^, Jiazhen Li^1,*^, Changliang Peng^3,*^

^1^Department of Orthopedic Surgery, The First Affiliated Hospital of Zhengzhou University, Zhengzhou, China

^2^Department of Magnetic Resonance Imaging, The First Affiliated Hospital of Zhengzhou University, Zhengzhou 450052, China

^3^Department of Spinal Surgery, The Second Hospital of Shandong University, Shandong University, Jinan 250033, China

**^†^Xinchang Lu and Xueping Zhang contributed equally to this work**

***Corresponding author**: Jiazhen Li, E-mail: jzhli6411@163.com;

Changliang Peng, E-mail: pcliang@sdu.edu.cn

**Supplementary Methods**

**Cell culture**

Human osteosarcoma (OS) cell lines (MG63 and143B) and the human osteoblast cell line (hFOB1.19) were purchased from Shanghai Biotechnology Co., Ltd. (Shanghai, China). Human monocytic leukemia cell line THP-1, highly metastatic murine OS cell line (K7M2 wild-type-WT), and human umbilical vein endothelial cells (HUVECs, passage 3) were obtained from Wuhan Pricella Biotechnology Co., Ltd. (Wuhan, China). The 143B and THP-1 monocytes were cultured in RPMI-1640 medium (Gibco), while MG63, hFOB1.19, and K7M2 cells were grown in DMEM medium (Gibco). HUVECs were maintained in endothelial cell medium (ECM; ScienCell, Carlsbad, CA, USA). All media were supplemented with 10% fetal bovine serum (FBS, Gibco), 100 IU/mL penicillin G, and 100 μg/mL streptomycin. All cells were incubated in a humidified atmosphere of 95% air and 5% CO2 within a CO2-regulated incubator. All cell lines were authenticated using short tandem repeat (STR) profiling and tested negative for mycoplasma contamination before use in experiments.

**Reverse transcription-quantitative polymerase chain reaction (****RT-qPCR)**

Total RNA was isolated using TRIzol reagent (Invitrogen) following the manufacturer's protocol. Complementary DNA (cDNA) synthesis was performed with the GoScript™ Reverse Transcription System kit (Promega). Quantitative PCR (qPCR) was conducted on a Bio-Rad CFX96 Touch sequence detection system using ChamQ SYBR qPCR Master Mix (Vazyme, Biotech Co., Ltd). GAPDH served as the internal reference gene for relative quantification of gene expression, analyzed using the 2^-ΔΔCT^ method. The primers used in the sequences are listed in online Supplementary Table 1. All primers were synthesized by Beijing Qingke Biotechnology Co., Ltd. (Beijing, China).

**Western blotting**

Following cell collection, the cells were lysed using RIPA buffer (Beyotime, Shanghai, China) combined with a protease inhibitor cocktail (Roche, Basle, Switzerland) and a phosphatase inhibitor (Beyotime, Shanghai, China). Following separation on SDS/PAGE gels, protein samples were put onto PVDF membranes (Millipore, Burlington, MA, USA). After blocking the membrane for one hour at room temperature with a 5% skim milk powder in 5% Tween-20 TBST solution, the membrane was incubated with the corresponding antibody for the entire night at 4°C. Following three TBST washes, the secondary antibody was used to probe the membrane. The membrane was probed using the ECL system (Bio-rad ChemiDoc) following three further TBST washes. The antibodies used are listed in online Supplementary Table 2.

**Lentiviral vectors construction** **and transfection**

All plasmids utilized in this investigation were cloned by VectorBuilder (Guangzhou, China). The whole human SPON2 cDNA was inserted into the lentiviral vector pLV[Exp]-EGFP/Puro-EF1A to create SPON2-overexpression lentiviral vectors (oe-SPON2). To deplete SPON2, a human SPON2-targeting shRNA sequence was inserted into the lentiviral vector pLV[shRNA]-EGFP/Puro-U6, resulting in SPON2-RNAi lentiviral vectors (sh-SPON2). The sequence is 5′-GCUGGGACAGAAAGUGTUAAU-3′. A scrambled control shRNA was designed and synthesized by VectorBuilder to serve as a negative control (sh-NC) in the experiments. Lentiviral generation and infection were conducted in accordance with the manufacturer's guidelines. Cells exhibiting sustained lentiviral expression were subjected to selection for 10 days using 0.5 μg/mL puromycin (Thermo Fisher Scientific), and the efficacy of SPON2 knockdown or overexpression was validated using RT-qPCR and western blotting.

**Wound healing assay**

Transfected cells (5 × 10⁵) were cultured in 6-well plates until near confluence and thereafter incubated in serum-free media for 24 h. Linear incisions were made in the cell monolayers using 200 μL pipette tips, followed by two washes with phosphate-buffered saline (PBS). The photos were obtained at 0 h and 24 h utilizing a microscope (Leica, Wetzlar, Germany).

**Transwell assay**

The transwell assay was performed to evaluate cell migration and invasion. Transfected cells (2 × 10⁵) were resuspended in 200 µL of serum-free medium and seeded into the upper chambers of transwell inserts (8 µm pore size; Corning, NY, USA) pre-coated with Matrigel (BD Biosciences). The lower chambers were filled with medium supplemented with 10% FBS as a chemoattractant. After 24 h of incubation, cells that had migrated to the lower surface of the membrane were fixed with methanol, stained with Crystal Violet Solution (Beyotime, Shanghai, China), and visualized using an inverted microscope. Images were captured to document the results.

**Cell cycle analysis**

Cells were seeded in a 6-well plate at a density of 2 × 10^5^ per well. Afterward, cells were transfected with either sh-NC or sh-SPON2 and incubated for 48 h. Following transfection, OS cells were treated with or without lipopolysaccharide (LPS, L3024, Sigma-Aldrich, USA) at a final concentration of 1 µg/mL—an empirically established dosage widely used in cancer-related in vitro models to activate NF-κB signaling without inducing cytotoxicity, as described in the main text. Then, cell flow cytometry was employed to analyze the cell cycle of cells. Cells were collected and fixed in 70% ethanol at 4 °C overnight, subsequently stained with propidium iodide (PI), and analyzed using a FACS flow cytometer (Becton-Dickinson, Mountain View, CA). Each sample underwent analysis of 20,000 cells using CellQuest software (Becton Dickinson). The distribution of the cell cycle was studied, and cells in the G1, S, or G2/M phases were quantified using ModFit software.

**Tube formation assay**

The experimental groups are the same as those described in the methods for the cell cycle analysis. Briefly, 150 µL of growth factor-reduced Matrigel (BD Biosciences) was thawed overnight on ice at 4 °C, then evenly distributed into a 24-well plate and incubated at 37 °C for 30 minutes to solidify. Cells from various experimental groups were harvested using trypsin digestion, resuspended in serum-free DMEM, counted, and adjusted to a final concentration of 1×10⁵ cells/mL. Subsequently, 5×10⁴ cells were seeded into each well pre-coated with Matrigel. After 72 h incubation, tube-like structures were visualized and captured using microscopy. The number of tubes formed in each well was quantified using ImageJ software.

**Analysis of cell morphology and epithelial-mesenchymal transition (EMT) induction**

Cells were transfected with sh-NC or sh-SPON2, as described above, and incubated for 48 h. During this period, cells were treated with 50 ng/mL epidermal growth factor (EGF, BD Biosciences) twice, with each treatment lasting 1 h, to enhance the stimulatory effect of EGF. The cells were cultured in DMEM supplemented with 1% FBS to further optimize EGF responsiveness. Additionally, the cells were treated with or without 1 µg/mL LPS. The experimental groups were defined as follows: the control group consisted of untreated cells serving as the baseline control. In the EGF-treated group, cells were treated with 50 ng/mL EGF. For the EGF + sh-NC group, cells were transfected with sh-NC and treated with 50 ng/mL EGF. The EGF + sh-SPON2 group included cells transfected with sh-SPON2 and treated with 50 ng/mL EGF. Finally, the EGF + sh-SPON2 + LPS group comprised cells transfected with sh-SPON2, treated with 50 ng/mL EGF, and additionally treated with 1 µg/mL LPS. Cell morphology was analyzed through microscopic examination to assess structural changes.

In addition to morphological examination, EMT marker detection was performed by transfecting cells with sh-NC or sh-SPON2, or treating them with 1 µg/mL LPS. After 48 h, cells were collected through trypsin digestion for further analysis. EMT marker expression was evaluated using RT-qPCR and western blotting.

**Induction of macrophage polarization**

To induce M0 macrophage, THP-1 cells were seeded in a 6-well plate at a density of 1 × 10^6^ cells per well and stimulated with 100 ng/mL phorbol 12-myristate 13-acetate (PMA; MCE, Shanghai, China) at 37 °C and 5% CO2 for 48 h to facilitate their differentiation into adherent macrophages. Thereafter, floating cells were carefully removed, and the cells were exposed to 20 ng/mL interleukin-4 (IL4; MCE, Shanghai, China) for an additional 24 h to facilitate their polarization towards the M2 phenotype.

**Co-culture system**

MG63 cells were transfected separately with sh-NC or sh-SPON2 and incubated for 48 h. After transfection, the cells were treated with or without 1 µg/mL LPS for the specified duration. THP-1-derived macrophages were not exposed to LPS directly, but were co-cultured with LPS-treated or untreated OS cells. THP-1-derived macrophages and MG63 cells were co-cultured using a cell culture insert (Corning, New York, USA) with a 0.4 μm porous membrane to partition the upper and bottom chambers. Differentiated M2 macrophages (5×10⁵ cells/well) were incubated in the upper chamber, which was then placed into a 6-well plate pre-seeded with MG63 cells (1 × 10⁶ cells/well) transfected with sh-NC or sh-SPON2. The co-culture was maintained for 72 h, then cells were collected for further research.

**Macrophage marker identification**

RT-qPCR, western blotting, and flow cytometry were utilized to assess the polarization status of macrophages in the co-culture system. The mRNA levels of NOS2 (iNOS) and CD86 were analyzed as markers for M1 macrophages, while ARG1 and CD206 were examined as markers for M2 macrophages. Western blotting was conducted to measure the protein expression levels of CD206, MAC2 (Galectin-3), and CD68 in THP-1 cells derived from the co-culture system. For flow cytometry analysis, cells were harvested and resuspended in FACS tubes at a concentration of 10^6^ cells in 100 µL PBS. Subsequently, macrophages were labeled on ice for 30 minutes with 5 µL of specific primary antibodies against surface markers, including F4/80-PE and CD206-FITC (as an M2 marker). The stained cells were then washed twice with cold PBS, resuspended in 400 µL PBS, and analyzed for surface marker expression using a flow cytometer (Becton Dickinson). The antibodies used are listed in online Supplementary Table 2.

**Tumor xenograft assay**

Animal studies were approved by the Animal Ethical Committee of the First Affiliated Hospital of Zhengzhou University, and were conducted in accordance with the Management and Use Guidelines of Laboratory Animals of NIH. The stable cell line was created by transfecting K7M2 WT cells with lentiviral vectors expressing either the sh-SPON2 or the sh-NC. Female BALB/C nude mice, 5 to 6 weeks old, were acquired from Experimental Animal Center of Zhengzhou University (Zhengzhou, China). The mice were randomly divided into four groups, each with six mice: Control, sh-NC, sh-SPON2, and sh-SPON2+LPS. After the mice were anesthetized with isoflurane, 1 × 10^6^ K7M2 WT cells expressing sh-NC or sh-SPON2 were suspended in 20 μL of PBS and subcutaneously injected into the proximal tibia through the cortex of the anterior tuberosity using a 30-gauge needle. On the 7th day after tumor formation, the mice were injected twice weekly with 2.5 mg/kg of LPS or an equivalent amount of PBS until the day before the end of the experiment. Approximately two weeks later, a tumor volume of around 200 mm³ was attained. The dimensions of tumors (D1, D2) were assessed bi-daily using a caliper, and tumor volume was computed with the formula V = 4/3π[1/4(D1 + D2)]². After six weeks, lung metastases from mice injected with sh-NC or sh-SPON2 cells were assessed. The mice were euthanized under anesthesia, and tissues were collected. Tumor weights were measured using an electronic scale. Following euthanasia, the lungs were harvested, fixed in 4% paraformaldehyde, and embedded in paraffin. To quantify pulmonary metastatic lesions, the entire lungs were sectioned into sequential 4 μm-thick slices. These sections were stained with hematoxylin and eosin (H&E) and examined under a light microscope to identify metastases.

Total RNA or protein was isolated from tissues, then RT-qPCR was used to detect the gene expression levels of SPON2 in xenograft mouse tissues and the M1 macrophage markers NOS2 and CD86, as well as the M2 macrophage markers CD206 and Arg1. Western blotting was used to detect the protein expression of EMT-related proteins N-Cadherin, Vimentin, and E-Cadherin. Flow cytometry was used to detect the quantities of M1 macrophage phenotypes (CD68 and NOS2) and M2 macrophage phenotypes (CD68 and CD206) in tumor tissues. Immunohistochemistry (IHC) assay was used for the protein detection of Ki67 and SPON2 in tumors. Supplementary Table 2 summarizes the antibodies used.

**Flow cytometry analysis**

Macrophage phenotypes were characterized using flow cytometry based on the surface expression of specific markers, following established protocols. In brief, tissues were minced into small fragments using surgical scissors and homogenized to prepare a cell suspension. After centrifugation, residual erythrocytes were eliminated using an erythrocyte lysis solution. Lymphocytes were subsequently isolated with a lymphocyte separation solution. The harvested cells were washed with ice-cold PBS and adjusted to a concentration of 1 × 10⁶ cells/mL. The cell suspension was stained with antibodies targeting CD68-PE, NOS2-FITC, and CD206-FITC. Data acquisition was performed using a CytoFLEX flow cytometer, and results were analyzed with CytExpert 2.4.0 software (Beckman Coulter).

**IHC**

Briefly, paraffin-embedded xenograft mouse tissue sections were deparaffinized, rehydrated, and treated to inhibit endogenous peroxidase activity. Non-specific protein binding was blocked, and the sections were incubated overnight at 4°C with primary antibodies against Ki67 and SPON2. This was followed by a 30-minute incubation with secondary antibodies at room temperature. Staining was developed using the DAB Detection Kit (Beyotime, P0202). Images of the stained tumor sections were captured using the DAKO REAL EnVision Inspection System.

**Gene set enrichment analysis (GSEA)**

To investigate differences in gene expression associated with varying levels of SPON2 expression, gene expression profiles from 34 OS samples with metastasis and 19 OS samples without metastasis (GSE21257, https://www.ncbi.nlm.nih.gov/gds/) were analyzed. Genes significantly correlated with either high or low SPON2 expression were identified using the curated gene set collection from the Molecular Signatures Database (MSigDB) C2. GSEA was performed, and the results were presented as normalized enrichment scores (NES). Statistical significance was determined based on a false discovery rate (FDR) threshold of less than 0.25 and a P-value threshold of less than 0.05.

**Differential expression and prognostic analysis of SPON2 in pan‐cancer**

The pan-cancer dataset was obtained from the UCSC Xena database (<https://xenabrowser.net/>), and SPON2 expression data across cancer types were extracted. Differential expression between normal and tumor tissues was assessed using unpaired Wilcoxon rank-sum and signed-rank tests in R (version 3.6.4). Kaplan–Meier survival curves were generated via the Kaplan–Meier Plotter (<http://kmplot.com/analysis/>), with the optimal expression cut-off used for group stratification.

**Statistical analysis**

All quantitative data are expressed as mean ± standard deviation (SD). The Student’s t-test, Wilcoxon rank-sum test, and one-way ANOVA with Tukey’s post hoc test were employed to compare continuous variables between groups. Survival data were illustrated using Kaplan–Meier curves, and survival differences were assessed by the log-rank test. Statistical analysis was conducted using GraphPad Prism (version 8). A p-value of less than 0.05 was employed to denote statistical significance.
