# Supplementary material for: SPON2 facilitates osteosarcoma development by inducing M2 macrophage polarization through activation of the NF-κB/VEGF signaling axis
Source: Cell Death Discov. 2025 Jul 29;11:352. doi: 10.1038/s41420-025-02626-2 (PMC12307574; doi:10.1038/s41420-025-02626-2)
Supplement: Supplementary file 2 — Supplementary Table 1 [file 41420_2025_2626_MOESM2_ESM.docx]

**Supplementary Table 1. Primer sequences used in the study.**

|  | **Forward Primer (5′→3′)** | **Reverse Primer (5′→3′)** |
| --- | --- | --- |
| **Used for**  **RT-qPCR** |  |  |
| *SPON2* | CTGGACCTGTACCCCTACGA | ACGCACTCAGCCTCTTCTTC |
| *Slug* | CCTGGTTGCTTCAAGGACAC | TCCATGCTCTTGCAGCTCTC |
| *Snail* | GAGGCGGTGGCAGACTAG | GACACATCGGTCAGACCAG |
| *Twist* | GTCCGCAGTCTTACGAGGAG | GCTTGAGGGTCTGAATCTTGCT |
| *ZEB1* | TGGCAAGACAACGTGAAAGA | AACTGGGAAAATGCATCTGG |
| *NOS2* | ATGGCCTGTCCTTGGAAATT | TCAGAGCGCTGACATCTCCA |
| *CD86* | TCTGCTGCTGTAACAGGGACTA | TAGGTTCTGGGTAACCGTGTAT |
| *CD206* | TGTATTCTTTGCCTTTCCCAGTCTC | CCTCAA AACAGACTTACCCAATAGCTG |
| *ARG1* | AAGCAGACCAGCCTTTCTCA | GCCAAGTCCAGAACCATAGG |
| *GAPDH* | GGAGCGAGATCCCTCCAAAAT | GGCTGTTGTCATACTTCTCATGG |
