# Supplementary material for: SPON2 facilitates osteosarcoma development by inducing M2 macrophage polarization through activation of the NF-κB/VEGF signaling axis
Source: Cell Death Discov. 2025 Jul 29;11:352. doi: 10.1038/s41420-025-02626-2 (PMC12307574; doi:10.1038/s41420-025-02626-2)
Supplement: Supplementary file 3 — Supplementary Table 2 [file 41420_2025_2626_MOESM3_ESM.docx]

**Supplementary Table 2. Antibodies used in the study.**

| **Antibody** | **Company** | **Catalogue No.** | **Application** | **Dilution** |
| --- | --- | --- | --- | --- |
| **Used for Western blotting (WB)** | | | | |
| anti-SPON2 | Abcam | ab302897 | WB | 1:1000 |
| anti-JNK | Abcam | ab251542 | WB | 1:1000 |
| anti-p38 MAPK | Affinity | AF6455 | WB | 1:1000 |
| anti-Phospho-JNK | CST | 9251S | WB | 1:1000 |
| anti-Phospho-p38 MAPK | Abcam | ab170099 | WB | 1:1000 |
| anti-Bcl-2 | Abcam | ab32124 | WB | 1:1000 |
| anti-Bcl-xL | Abcam | ab98143 | WB | 1:1000 |
| anti-Mcl-1 | Abcam | ab28147 | WB | 1:1000 |
| anti-VEGF | Abcam | ab106580 | WB | 1:1000 |
| anti-RANK | Abcam | ab182158 | WB | 1:1000 |
| anti-RANKL | Abcam | ab9957 | WB | 1:1000 |
| anti-MMP2 | Abcam | ab92536 | WB | 1:1000 |
| anti-MMP9 | Abcam | ab76003 | WB | 1:1000 |
| anti-N-Cadherin | Abcam | ab76011 | WB | 1:1000 |
| anti-Vimentin | Abcam | ab137321 | WB | 1:1000 |
| anti-E-Cadherin | Abcam | ab308347 | WB | 1:1000 |
| anti-NF-κB p65 | Abcam | ab32536 | WB | 1:1000 |
| anti-Phospho-NF-κB p65 | Abcam | ab76302 | WB | 1:1000 |
| anti-IL10 | Affinity | DF6894 | WB | 1:1000 |
| anti-CCL2 | Affinity | BF0556 | WB | 1:1000 |
| anti-CSF1 | Affinity | BF0475 | WB | 1:1000 |
| anti-CD206 | Proteintech | 81525-1-RR | WB | 1:1000 |
| anti-MAC2 | Proteintech | 14979-1-AP | WB | 1:1000 |
| anti-CD68 | Proteintech | 28058-1-AP | WB | 1:1000 |
| anti-GAPDH | Abcam | ab245355 | WB | 1:5000 |
| HRP Goat Anti-Rabbit IgG | Abcam | ab6721 | WB | 1:5000 |
| HRP Goat Anti-Mouse IgG | Proteintech | SA00001-1 | WB | 1:5000 |
| **Used for Flow Cytometry (FCM) and immunohistochemical (IHC) staining** | | | | |
| PE Anti-F4/80 | Thermo | 12-4801-82 | FCM | 1:50 |
| FITC-Anti-CD206 | Thermo | MA5-16870 | FCM | 1:50 |
| PE Anti-CD68 | Thermo | MA5-16653 | FCM | 1:50 |
| PE Anti-CD86 | Thermo | MHCD8604 | FCM | 1:50 |
| FITC-Anti-NOS2 | Santa Cruz | sc-7271 | FCM | 1:50 |
| anti-SPON2 | Abcam | ab215451 | IHC | 1:100 |
| anti-Ki67 | Abcam | ab15580 | IHC | 1:200 |
