# Supplementary figures and images for: SPON2 facilitates osteosarcoma development by inducing M2 macrophage polarization through activation of the NF-κB/VEGF signaling axis
Source: Cell Death Discov. 2025 Jul 29;11:352. doi: 10.1038/s41420-025-02626-2 (PMC12307574; doi:10.1038/s41420-025-02626-2)

Fig.1B

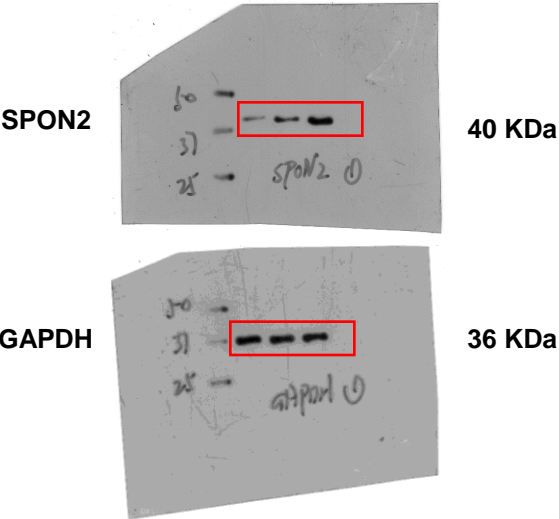

Fig.2C

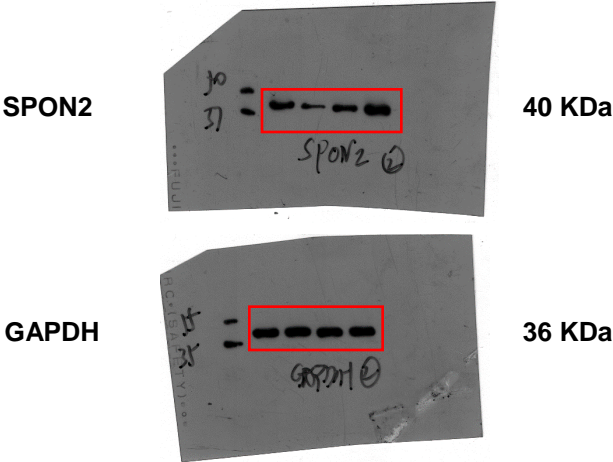

**Fig. 3**

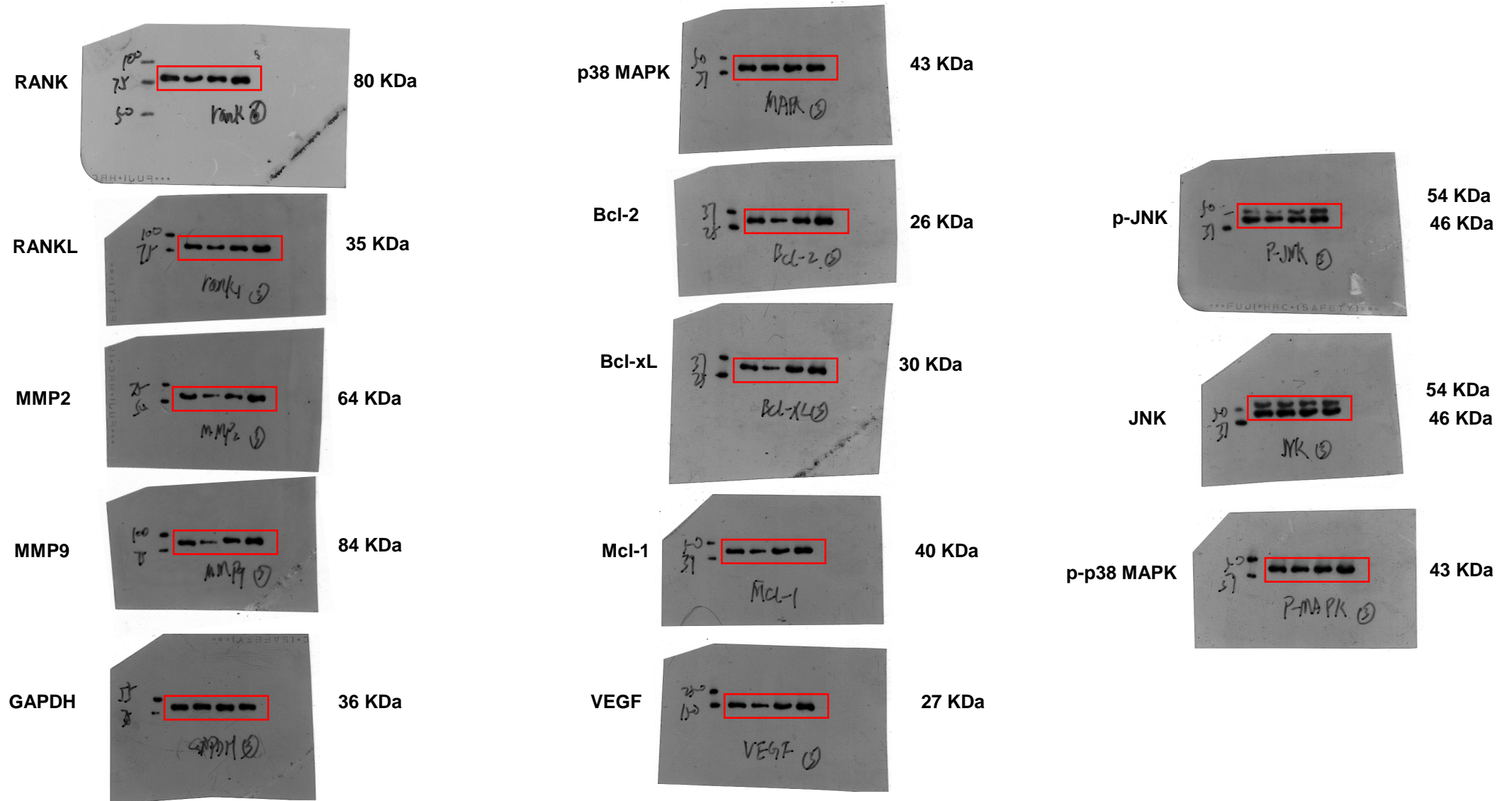

**Fig. 4E**

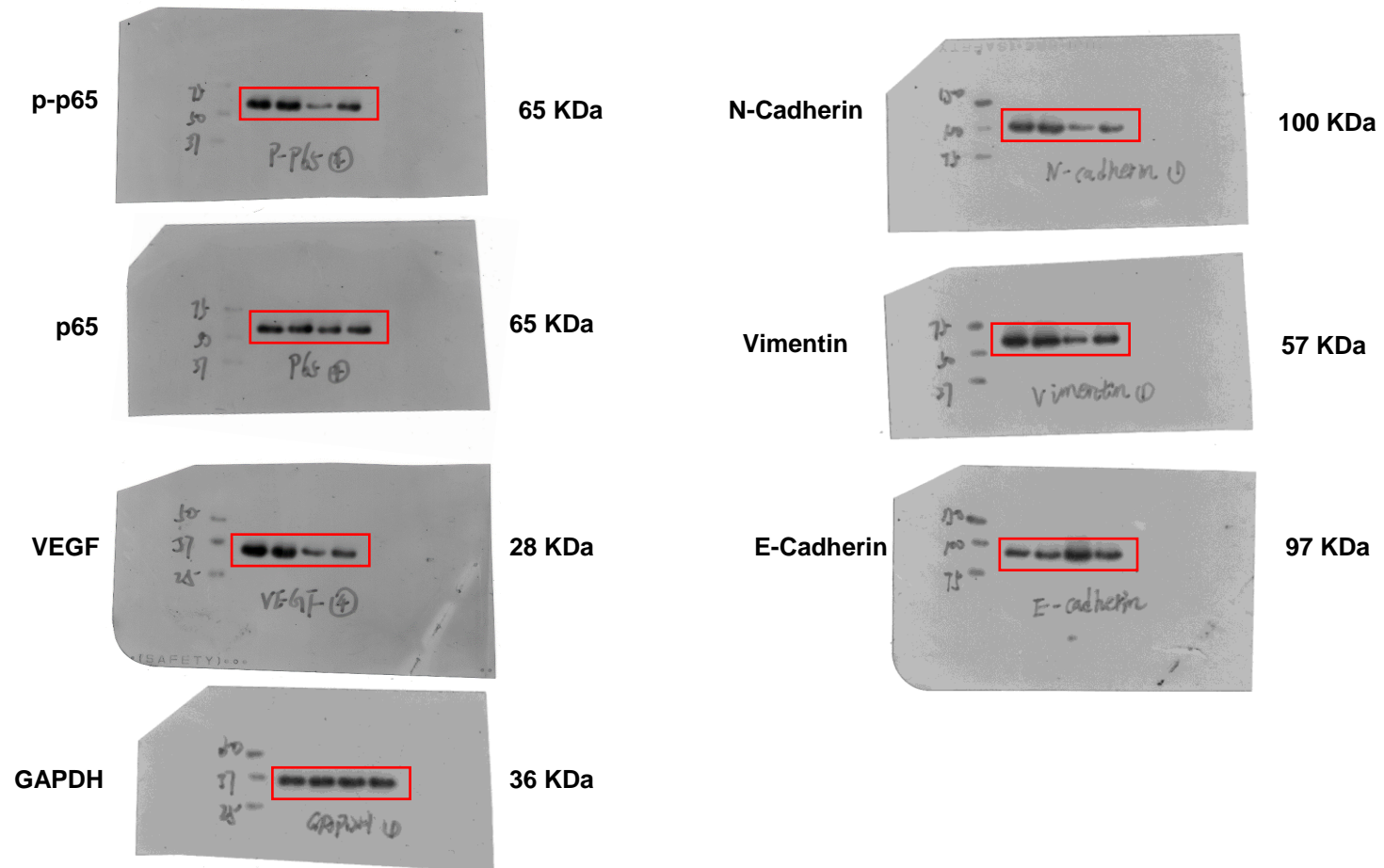

**Fig. 5A**

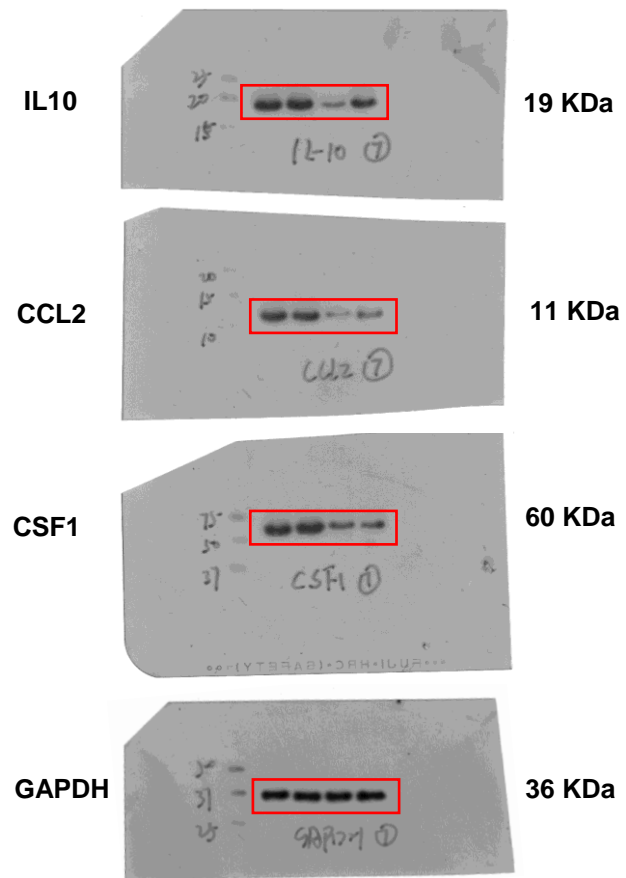

**Fig. 5C**

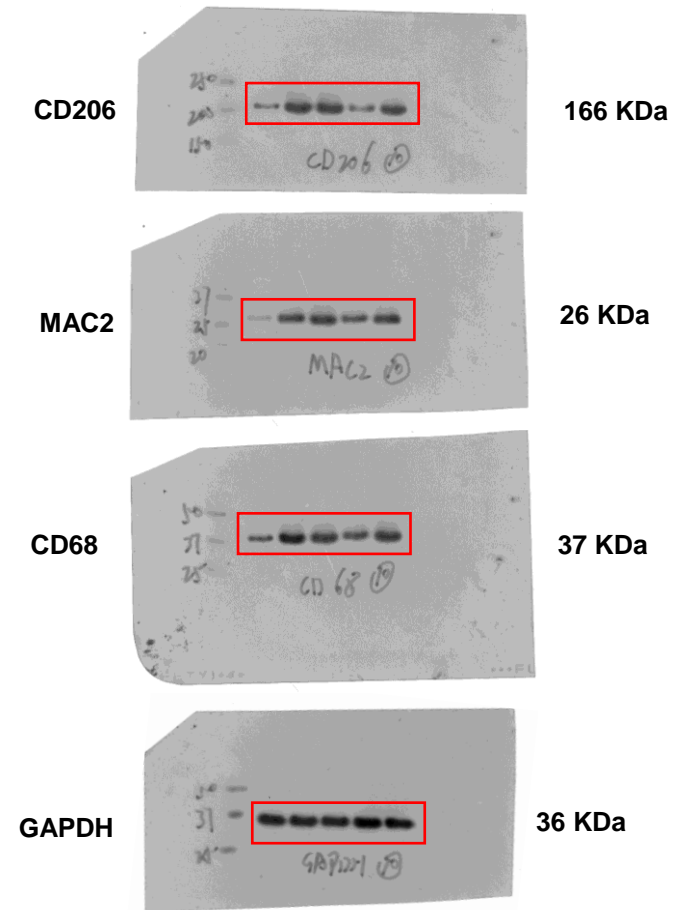

Fig. 7C

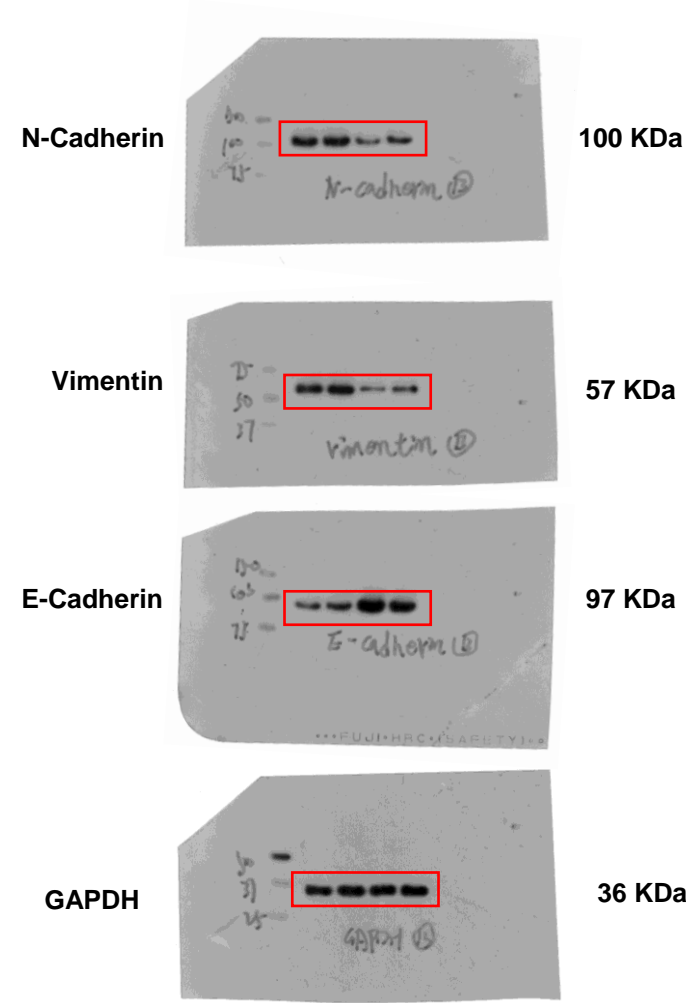

Fig. 7D

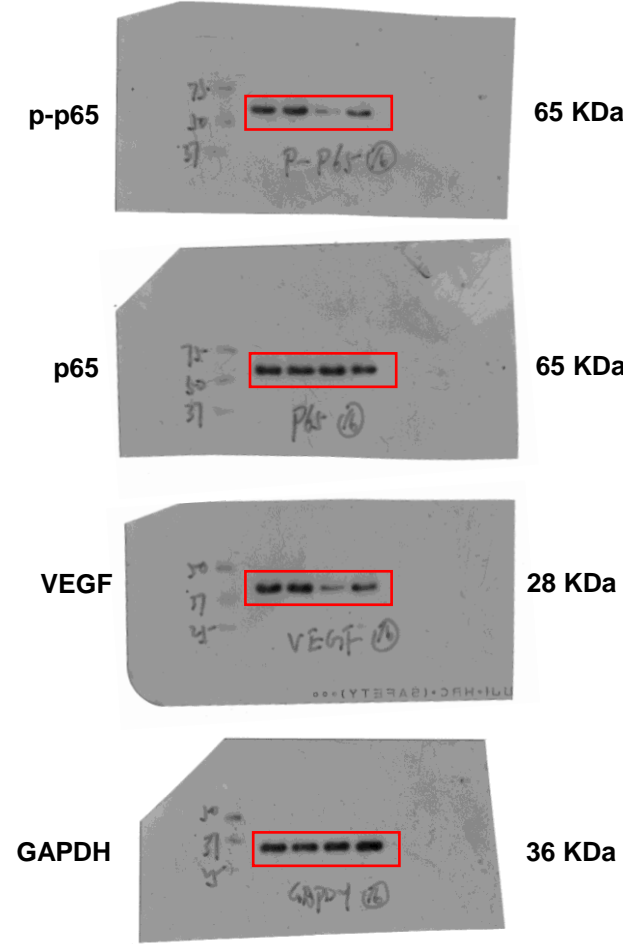

Supplement: Supplementary file 4 — Original Western blot file [file 41420_2025_2626_MOESM4_ESM.pdf]
